# Microfluidic active loading of single cells enables analysis of complex clinical specimens

**DOI:** 10.1038/s41467-018-07283-x

**Published:** 2018-11-14

**Authors:** Nicholas L. Calistri, Robert J. Kimmerling, Seth W. Malinowski, Mehdi Touat, Mark M. Stevens, Selim Olcum, Keith L. Ligon, Scott R. Manalis

**Affiliations:** 1 Koch Institute for Integrative Cancer Research, Massachusetts Institute of Technology, Cambridge, MA USA; 2 Department of Oncologic Pathology, Dana-Farber Cancer Institute, Boston, MA USA; 3 Department of Medical Oncology, Dana-Farber Cancer Institute, Boston, MA USA; 4 Department of Pathology, Harvard Medical School, Boston, MA USA; 5 Department of Pathology, Boston Children’s Hospital, Boston, MA USA; 6 Department of Pathology, Brigham and Women’s Hospital, Boston, MA USA; 7 Department of Biological Engineering, Massachusetts Institute of Technology, Cambridge, MA USA; 8 Department of Mechanical Engineering, Massachusetts Institute of Technology, Cambridge, MA USA

## Abstract

A fundamental trade-off between flow rate and measurement precision limits performance of many single-cell detection strategies, especially for applications that require biophysical measurements from living cells within complex and low-input samples. To address this, we introduce ‘active loading’, an automated, optically-triggered fluidic system that improves measurement throughput and robustness by controlling entry of individual cells into a measurement channel. We apply active loading to samples over a range of concentrations (1–1000 particles μL^−1^), demonstrate that measurement time can be decreased by up to 20-fold, and show theoretically that performance of some types of existing single-cell microfluidic devices can be improved by implementing active loading. Finally, we demonstrate how active loading improves clinical feasibility for acute, single-cell drug sensitivity measurements by deploying it to a preclinical setting where we assess patient samples from normal brain, primary and metastatic brain cancers containing a complex, difficult-to-measure mixture of confounding biological debris.

## Abstract (web summary)

Single-cell detection methods are limited by the trade-off between flow rate and measurement precision. Here the authors introduce active loading, an optically triggered microfluidic system to concentrate diluted cell samples, which reduces clogging and decreases processing time in single-cell assays.

## Introduction

The high level of control offered by microfluidic devices has proven to be valuable for single-cell biological assay development, where measurement of individual cells or small clusters of cells can now be performed with exquisite fidelity. However, for platforms that incorporate on-chip detection, flow rate is governed by the bandwidth required for the measurement, which imposes limitations on the maximum achievable throughput. Although measurements such as fluorescent intensity or light scattering can approach 10^5^ cells s^−1^, biophysical methods such as spectroscopy^1,2^, deformability^3–7^, and electrical impedance^8^ typically require bandwidths in the 0.1 Hz to 10 kHz range, limiting throughput to the range of 1–10,000 cells min^−1^ (Supplementary Table 1). Throughput for these approaches can be raised by increasing concentration; however, there are often biological and logistical factors that determine the range of achievable sample concentrations. For example, samples processed from primary tissue sources—including biopsies, fine-needle aspirates, blood samples, patient-derived xenograft tissues, and so on—often yield a limited number of cells of interest that set inherent limits on the maximum achievable sample concentration. Additionally, the loading period of particles into a device is limited by Poisson statistics and flow rate, which makes dilute samples especially challenging without increasing flow rate and sacrificing bandwidth.

To decouple this fundamental trade-off between flow rate into the device and measurement bandwidth, we developed an approach called “active loading” where a triggering detector selectively isolates particles from a large, two-port sampling channel into a second smaller measurement channel. Since the flow rates in each channel can be independently controlled, it is possible to set the flow rate in the measurement channel based on the desired measurement bandwidth while dynamically controlling the sampling channel flow rate in order to deterministically load particles into the measurement channel. Using bright-field microscopy as the triggering detector and standard pressure-driven fluidic control components, we improve the throughput for a particle concentration of 50 μL^−1^ by over 10-fold without changing the measurement bandwidth. By applying active loading to the serial suspended microchannel resonator (sSMR), we show that buoyant mass and growth properties can be measured from a dilute concentration of only a few cells per microliter in 3 h. In contrast, the same number of measurements would take over 3 days of continuous passive sampling. A key advantage of active loading with imaging is that debris can be rejected in order to reduce clogging and eliminate unnecessary measurement time. We demonstrate this capability by measuring the drug sensitivity from a range of clinical brain tissue and tumor resection samples containing a complex mixture of confounding biological debris after cell purification.

## Results

### Active loading

**Fig. 1:**
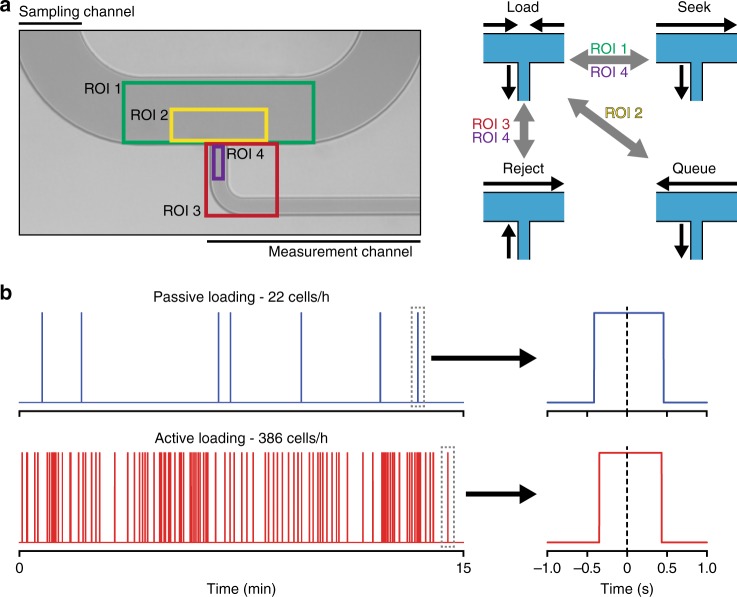
Schematic of active loading by optically triggered fluidic state switching. **a** Regions of interest (ROIs) are labeled as colored boxes. ROI 1 (green) is used to detect particles when in the “seek” state. Detection of a particle at traveling at a high flow rate in the sampling channel by ROI 1 causes a temporary change to the default “load” state, and reverts following entrance of a single particle into the measurement channel as detected by ROI 4 (purple). ROI 2 (yellow) maintains the presence of a single particle in the sampling channel for the next loading duty cycle. As a single particle is detected by ROI 2 while in the “load” state it triggers adoption of a “queue” state, which bumps the cell back in the sampling channel before reverting to the “load” state. This continues until the duty cycle is complete. ROI 4 (purple) and ROI 3 (red) work together to detect entrance into the measurement channel and the presence of debris or doublet events, respectively. Once ROI 4 detects entrance of a particle in the “load” state, ROI 3 quickly images the event, switching to the “reject” state if the particles geometry or contrast is outside previously set parameters defining an unwanted particle. **b** Comparison between passive throughput (22 cells h^−1^, 95% CI: 13, 39, *n* = 9) and active loading (386 cells h^−1^, 95% CI: 354, 433, *n* = 247) for murine L1210 cells (50 μL^−1^) flowing through a transit time detector in the measurement channel (Supplementary Fig. 1, see Methods). Zoom-in plots show passage of a single cell with a predefined transit time of ~800 ms

**Fig. 2:**
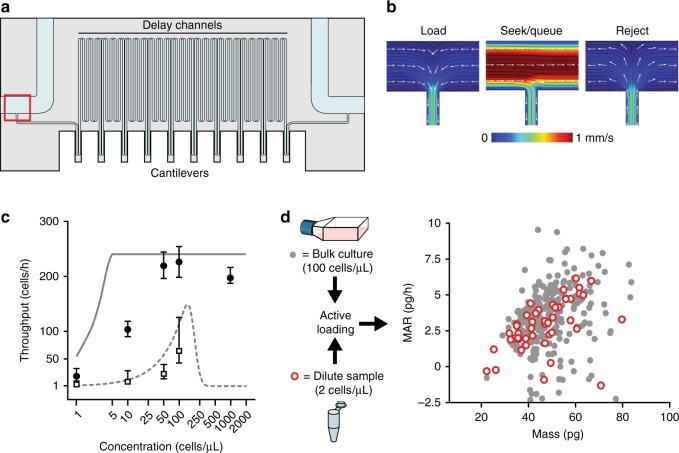
Active loading enables single-cell growth measurements of dilute samples. **a** Schematic of the serial suspended microchannel resonator (sSMR). Sampling channels on either side of the device (100 μm wide and 30 μm deep) are each accessed via two ports with independent pressure control to achieve the fluidic states presented in **b**. These sample channels are connected with a serpentine channel (50 cm long, 20 μm wide, and 25 μm high) with 10–12 SMR mass sensors spaced evenly along its length. Mass accumulation rate (MAR) is calculated by taking the slope of the linear least squares fit of mass measurements collected from individual SMRs as a function of time for each single-cell trajectory. **b** COMSOL models demonstrating the flow characteristics of the four different fluidic states presented in Fig. 1a. The model shows the T-junction entrance of the sSMR, outlined with a red box in **a**. Flow patterns were modeled using the volumetric flow rates described in Supplementary Note 4 to recapitulate experimental conditions. **c** Comparison of theoretical throughput limits (solid and dashed lines for active and passive loading, respectively) with experimental results (solid points and open squares for active and passive loading, respectively) for samples with 1, 10, 50, 100, and 1000 L1210 cells μL^−1^ (*n* = 15, 105, 143, 149, and 83 for active loading and *n* = 1, 8, 64, 87, and 309 for passive loading) collected with a 15 s minimum spacing. The theoretical model is based on a 15 s duty cycle (Supplementary Note 4). Measurement error bars represent the 95% CI (two-tailed *t* test) of loading period (s) converted to throughput (events h^−1^). Each concentration was measured continuously for at least 20 min. The passive loading sample at 1000 cells μL^−1^ had a throughput of 747 cells h^−1^, 95% CI: 673, 832. **d** Dot plot of MAR vs. mass comparing L1210 cells measured from standard, growth-phase culture concentrations (100 cells μL^−1^, gray circles, *n* = 426), or from samples with low concentration and low total cell count (~2 cells μL^−1^, 100 total cells, open red circles, *n* = 47)

Multiple regions of interest (ROIs) are used to detect particles within either the sampling or measurement channels to enable optically triggered activation of various fluidic “states” and isolate individual cells with a defined loading duty cycle (Figs. 1a, 2b, Supplementary Note 1). The baseline state of the system is a “load” state, which is functionally equivalent to the passive fluidic approach, where the upstream and downstream pressures applied to the sampling channel are equal and a fixed pressure drop is maintained across the measurement channel, thereby setting the average transit time (and the required minimum bandwidth) for individual particles across the detector within the measurement channel. In this state, the volumetric flow into the sampling channel is identical to the flow in the measurement channel and therefore particles are loaded into the measurement channel in a strictly concentration-dependent manner governed by Poisson statistics.

In order to rapidly isolate particles from a dilute sample, the system toggles to a “seek” state. For this task, a pressure drop is applied along the sampling channel to induce a larger volumetric flow rate. During this adjustment, the pressure drop along the measurement channel is unchanged in order to maintain a constant flow rate to ensure consistent single-particle transit time through the detector. The flow along the sampling channel continues until a particle is detected in ROI 1, at which point the system switches to the “load” state to capture the particle in the measurement channel (Supplementary Movie 1). Since the sampling channel and measurement channel flow rates are largely decoupled, the maximum sampling channel flow rate is limited by the frame rate of the camera used for detection (Supplementary Notes 3, 4).

To maximize throughput, it is important to identify the next particle available to be measured. To achieve this, the user sets a loading duty cycle that maximizes loading throughput while maintaining the desired measurement bandwidth. Once a particle has entered the measurement channel (as detected by ROI 4), the system repeats the “seek” function. However, the next particle may be detected by ROI 1 prior to completion of the defined loading duty cycle. This occurs for dilute samples where the next particle is not immediately available but is found quickly by the “seek” function as well as high-concentration samples where multiple particles may be proximal to the measurement channel. In order to ensure that no more than one particle is loaded per duty cycle, the system adopts a “queue” state when a cell reaches ROI 2, but the loading duty cycle is not yet complete (Supplementary Movie 2). The “queue” state is characterized by a brief flush of the particle upstream by introducing a pressure drop along the sampling channel, at which point the system returns to the “load” state. This function repeats as necessary to keep the particle proximal to the measurement channel entrance until sufficient time has elapsed, at which point it is immediately loaded into the measurement channel. This “queue” state, combined with detection in seek mode, is key to enabling high throughput with evenly spaced particle sampling that is not reliant on Poisson statistics.

Finally, to determine if a particle loaded into the measurement channel is a particle of interest and not debris that should be excluded from measurement, the system implements a function driven by real-time image processing (Supplementary Movie 3). This process relies on user-defined thresholds for particle parameters such as cross-sectional area and *x*–*y* ratio (Supplementary Note 2). When a particle is loaded into the measurement channel, as detected by ROI 4, ROI 3 captures a bright-field image that is assessed for these parameters. If an undesired particle is loaded, a “reject” state is enabled whereby the pressure drop along the measurement channel is briefly reversed in order to remove the particle (Supplementary Movie 4, Supplementary Movie 5). At the same time, a pressure drop is induced along the sampling channel to flush this particle downstream and ensure that it is not recaptured for measurement. This feature allows for the rejection of debris loading events that would otherwise lead to failed measurements and enables high-fidelity measurements on samples with prohibitive amounts of biological debris or aggregates.

To demonstrate active loading, we used the first mass sensor of an sSMR to measure transit time of a murine lymphocytic leukemia cell line (L1210) at a concentration of 50 μL^−1^ (Fig. 1b, Supplementary Figure 1, Methods section). For passive loading, only 22 cells h^−1^ were measured for a desired transit time of 800 ms, while for active loading, 386 particles h^−1^ were measured without altering the transit time.

### Seek and queue functions increase concentration dynamic range

To demonstrate the benefits of active loading for a single-cell assay, we applied it to the sSMR for measuring mass accumulation rate (MAR)^9^. The sSMR is well suited for active loading since the sensor transit time is slow (typically ~600–800 ms) and coincidence within the long (~50 cm) measurement channel limits the maximum sample concentration (Fig. 2a, b, Supplementary Figure 1). We first determined the theoretical ranges of the concentration-dependent throughput for the sSMR with active and passive fluidic implementations (Supplementary Note 4). For passive loading, throughput increases for higher concentration samples before reaching a maximum theoretical throughput at an optimal cell concentration. Above this concentration threshold—which is defined by the minimum time required between cells flowing through the sSMR—cell matching failures begin to occur more frequently and the measurement throughput decreases. When this limitation is included, the active loading scheme displays a higher theoretical measurement throughput across all sample concentrations. For dilute-cell samples, this throughput advantage is driven largely by the “seek” function, whereas for medium and high-concentration samples it is driven largely by the “queue” functionality, which ensures sufficient spacing between cells to maintain cell matching fidelity and prevent co-occupancy of the measurement sensors.

These theoretical throughput improvements were tested experimentally by collecting single-cell MAR measurements for L1210 cells seeded at various concentrations (Fig. 2c). For high-concentration samples (above ~50 cells μL^−1^), we found that the system performed near the theoretical maximum throughput. For samples of moderate concentration, the advantage of active loading is particularly pronounced: for a sample concentration of 10 cells μL^−1^, the throughput increased from eight cells per hour for passive fluidic loading to ~100 cells h^−1^ using active loading.

To demonstrate the utility of the cell-seeking functionality, we collected single-cell MAR measurements for a sample containing approximately 100 L1210 cells in 50 μL of media (2 cells μL^−1^) (Fig. 2d). Over the course of a 3-h experiment, 47 of these cells were isolated for measurement, a data set that would have required approximately 21 h to collect with passive loading. Furthermore, the fluidic manipulation necessary to conduct this cell-seeking routine did not appear to introduce excessive stress on the cells as there were no significant differences in mass or MAR measurements observed as compared to L1210 cells measured with passive loading (Fig. 2d). In an analogous experiment using a 100 μL sample with approximately 270 hematopoietic cells (2.7 cells μL^−1^) from a murine pro-B cell line (BaF3), we collected 165 MAR measurements over 3 h (Supplementary Fig. 2). With passive loading, this experiment would have taken >3 days, which would have impacted cell growth dynamics, emphasizing the relevance of substantial throughput gains that are possible with active loading for devices where sampling and measurement flow rates are constrained.

Despite orders of magnitude throughput improvements demonstrated for dilute-cell samples, the throughput did not reach the theoretical limit depicted in Fig. 2c. This is due to non-zero response times of the pneumatic controls, which occasionally causes a cell detected in ROI 1 (Fig. 1a) to overshoot the measurement channel entrance. This overshoot is corrected with a brief flow reversal in the sampling channel, a process that slightly increases the average time between cell loading events (Supplementary Note 3).

### Rejection function reduces clogging from debris

A number of confounding factors preclude microfluidic technologies from being able to analyze single cells from heterogeneous patient biopsy samples. First, the number of cells that one can isolate from samples is highly variable, and often limited by either the biopsy sample size or isolation protocols. Additionally, primary samples generally present with a high level of biological debris and particulate aggregation, which limit flow rate by clogging the fluidic channels^10^. Sample debris and aggregation issues may be further exacerbated by ex vivo drug treatment of primary cells given that sensitive cells may undergo necrosis or apoptosis leading to fragmentation (mechanism dependent).

**Fig. 3:**
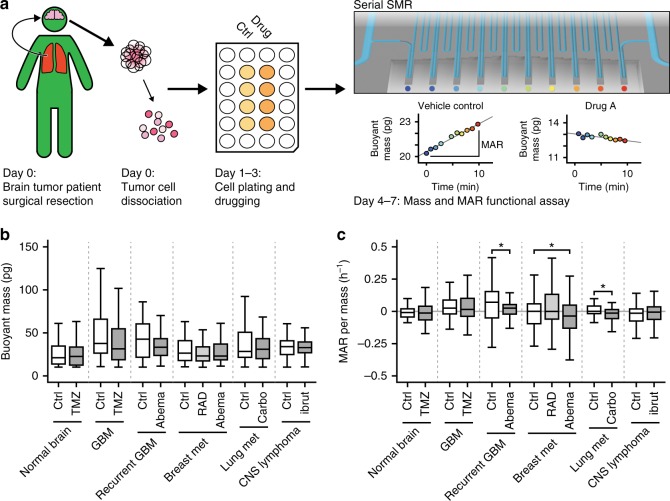
Ex vivo drug sensitivity testing of patient resections. **a** Sample processing pipeline for sSMR measurement with active loading. Tumor cells were isolated from patient resection specimens using established protocols^12^ (see Methods, Supplementary Note 7) for dissociation into single-cell suspension and allowed to recover for at least 24 h before the addition of drug or vehicle control. On subsequent days, the buoyant mass and MAR were measured for both the control and drug-treated fractions. **b** Tukey's box plot showing the buoyant mass measurements for primary biopsies of different brain lesions. From left to right, number of cells measured: *n* = 86, 90, 63, 64, 66, 83, 74, 60, 47, 53, 54, 164, and 188. **c** Tukey's box plot showing mass-normalized MAR values from the same primary tissue samples shown in **b**. Statistically significant reductions in MAR per mass (*p < 0.05 in highlighted segments) were observed for the recurrent glioblastoma treated with 1 μM abemaciclib for 72 h (*p* = 0.032), breast metastasis treated with 100 nM abemaciclib (*p* = 0.029), and lung metastasis treated with 100 μM carboplatin (*p* = 0.025). All other drug-control comparisons did not show a statistically significant response. Additional information about the handling of each primary sample can be found in Supplementary Note 7 and exact *p* values can be found in Supplementary Notes 9–14. For both **b**, **c**, the center line shows median value, hinges represent the first and third quartiles, and whiskers extend to the furthest value <1.5× IQR from hinge

Prior work demonstrates the capacity of MAR to define the therapeutic response of multiple myeloma patients to standard-of-care therapies^11^; however, solid tumors have remained difficult to measure. To determine whether active loading improves the feasibility of single-cell measurements on heterogeneous primary patient, we deployed sSMR devices with active loading to a preclinical laboratory setting at Dana-Farber Cancer Institute. Using established protocols for isolating single cells from primary tissue samples^12^ (Fig. 3a, Methods section, Supplementary Note 7), active loading enabled the sSMR to measure cell mass and MAR for a diverse range of clinical brain tissue and cancer samples exposed to either a standard-of-care therapy or experimental therapy currently in clinical trial (Supplementary Note 8). Measurements were obtained from five types of primary patient sample types including non-tumor brain tissue resected for a non-tumor condition (*n* = 1), primary and recurrent glioblastoma^13,14^ (*n* = 2), metastatic breast adenocarcinoma^15^ (*n* = 1), metastatic non-small-cell lung cancer^16^ (*n* = 1) and primary central nervous system (CNS) lymphoma^17^ (*n* = 1) (Fig. 3b,c, Supplementary Notes 9–14). Measurements were made in a median time frame of 9 days following surgery (range of 2–18 days). Overall, we were able to measure mass and MAR from 1092 cells with an average of 84 cells measured per condition over 13 conditions tested. (Fig. 3b, c). The buoyant mass, MAR, and mass-normalized MAR of each drug-treated sample were compared with a paired vehicle control and significance was calculated using the Wilcoxon's signed-rank test.

The “rejection” capability of active loading was essential in performing sSMR measurements on the primary biopsies, as they contained a high amount of undesirable debris and cell aggregates that could prematurely terminate measurements by clogging the measurement channel. All six primary samples had images recorded and annotated of every particle accepted or rejected by the real-time Labview code. These images were manually reviewed and compared with the real-time determination to quantify the success rate at identifying unwanted particles in real time (Supplementary Note 5). For the six primary samples measured, the overall success rate for the real-time analysis code was 86% for correctly identifying single cells and allowing them to continue through the measurement channel.

No change in mass nor MAR was observed in cells isolated from the normal brain treated with TMZ (250 μM, 72 h). Normal brain tissue is non-proliferative, and was used as a negative control for both drug response and baseline in vitro growth. Similarly, no significant change was observed in the primary CNS lymphoma treated with ibrutinib (10 nM, 48 h), or the newly diagnosed glioblastoma treated with TMZ (250 μM, 8 days). Mass-normalized MAR was significantly reduced for the recurrent glioblastoma (*p* = 0.032) treated with abemaciclib (1 μM, 72 h), breast metastasis (*p* = 0.029) treated with abemaciclib (100 nM, 72 h), and the lung metastasis sample (*p* = 0.025) treated with carboplatin (100 μM, 72 h). Although this was not a directed study to investigate the effects of therapies on primary cells, active loading improved throughput and enabled measurement of previously incompatible tissues. Future studies are needed in order to determine if the mass and MAR biomarkers can predict individual patient response to standard-of-care and experimental therapies.

## Discussion

Although numerous methods exist for tissue dissociation and pre-enrichment (e.g. centrifugation, filtration, and magnetic-activated cell sorting (MACS)), they often yield imperfect sample purification by leaving behind significant biological debris or cellular aggregates that make it challenging to analyze or manipulate single cells within microfluidics. The active loading approach presented here improves throughput of single-cell assays by reducing clogging events from debris or aggregates and circumventing limitations imposed by Poisson statistics for loading cells into the measurement channel. For the preclinical studies shown in Fig. 3, we utilized MACS-based cell enrichment and debris depletion upstream of the sSMR assay and found that these samples were still not easily measured without real-time debris rejection enabled by active loading. Thus, active loading is intended to supplement these existing purification methods to enable live-cell measurements from minimally processed and low-input clinical samples. Although the sSMR was used here, active loading could be used to improve performance of other single-cell measurement platforms provided that optical hardware required for imaging can be accommodated. However, benefits from circumventing limitations imposed by Poisson statistics only become meaningful when the necessary measurement time is more than ~100 ms, which is often the case for biophysical measurements (Supplementary Note 3).

While the implementation described here utilizes bright-field imaging with a low-cost camera for label-free detection (Supplementary Note 6), future iterations of active loading could achieve higher throughput by triggering with faster cameras or utilize fluorescent intensity readout with a photo-multiplier tube (Supplementary Note 3). Additionally, beyond basic geometry-based particle identification used here, improved image-processing algorithms could be used to generate more stringent classification criteria to better exclude debris and isolate cells of interest. Given the rapidly increasing number of microfluidic devices and single-cell assays in development for medical use, these universal improvements should be a benefit to the broader community.

## Methods

### Image analysis

Live images are acquired using a monochrome camera (BFS-U3-13Y3M-C, FLIR). Custom software coded in LabVIEW 2017 (National Instruments) is used to analyze images in real-time and integrate the image feedback with automated pneumatic control. A standard computer equipped with a 2015 4-core CPU with 8 Gb of RAM was capable of analyzing at least 60 frames s^−1^ stably. Settings specific to the image-processing code were calibrated using a suspension of polystyrene beads (Duke Scientific, #4207A) prior to loading biological samples on the sSMR.

### Pneumatic control

The sSMR features four fluidic ports. These ports connect to pneumatically sealed satellite reservoirs containing media or sample in sterile secondary vials. Independent electronic pressure regulators (QPV1TFEE030CXL, Proportion Air) control the pressure within the reservoir, which drives flow across the sSMR. Regulators are supplied with 5% CO_2_ gas, and the microfluidic chip and satellite reservoirs are kept at 37 ℃ using custom aluminum heat exchangers to maintain incubator-like conditions.

### Sample preparation

All liquids were filtered with 0.2-μm filters prior to use in the PDMS device or in cell culture. L1210 (murine lymphocytic leukemia, 87092804-1VL, ECACC/Sigma-Aldrich) and BaF3 (murine pro-B, Riken BioResource Center) cells were cultured in RPMI-1640 with l-glutamine (11875-093, Gibco) with added 10% dialyzed fetal bovine serum (F0392-500 mL, Sigma), 25 mM HEPES (15630-080, Gibco), and 1% ABAM (15240-062, Gibco). Cells are prepared by centrifuging for 5 min at 200 x *g*, removing the supernatant, and resuspension in fresh pre-warmed complete RPMI as defined above. These cell lines were not tested for mycoplasma contamination or authenticated.

Patient-derived cells from six different types of brain tissues were assessed for drug sensitivity in the sSMR: non-tumor brain tissue from epilepsy surgery, glioblastoma, recurrent glioblastoma, breast metastasis, lung metastasis, and primary CNS lymphoma. Resected samples were obtained with patient consent to research (Brigham and Women’s Hospital, DF/HCC IRB-approved consent protocol 10-417) were enzymatically and physically dissociated using the Brain Tumor Dissociation Kit P (130-095-942, Miltenyi Biotec) and gentleMACS Dissociator (130-093-235, Miltenyi Biotec). Cells were cultured in Neurocult NS-A proliferation media (05702, Stemcell Technologies) containing 20 ng mL^−1^ epidermal growth factor (130-093-825, Miltenyi Biotec) and 10 ng mL^−1^ fibroblast growth factor (130-093-564, Miltenyi Biotec).

After at least 48 h in culture (with the exception of CNS lymphoma which was cultured for 24 h), persistent red blood cells were removed with RBC lysis buffer (00-433-57, Thermo Fisher Scientific). The remaining cells were then dissociated with Accutase (A6964, Sigma-Aldrich) and further purified via demyelination (130-096-733, Miltenyi Biotec) with mass spectrometry separation columns (130-042-201, Miltenyi Biotec), or debris removal (130-109-398, Miltenyi Biotec). The purified cells were plated in 6-well or 24-well plates and allowed to recover in the well plate for 48–96 h before addition of the drug. Specific timelines in culture and drugging regimens for each tissue type can be found in Supplementary Note 7. Prior to loading samples on the sSMR for drug response measurements, cells were dissociated into a single-cell suspension using Accutase and gentle pipetting. Cells were resuspended at a concentration of 100,000 cells mL^−1^ in Neurocult NS-A (as prepared above) with the same concentration of drug or DMSO as their respective culture.

### Device preparation

The sSMR is cleaned prior to each experiment with 10% bleach for 10 min, followed by a 20-min rinse with DI-H_2_O. Persistent biological debris is removed with 0.25% Trypsin-EDTA. After cleaning, the device is passivated with 1 mg mL^−1^ PLL-g-PEG in H_2_O for 10 min at 37 ℃.

### SMR measurements for transit time detection

To detect cells and characterize transit time in Fig. 1, resonant frequency data was collected from the first cantilever of a sSMR (Supplementary Figure 1). Savitsky–Golay and nonlinear high-pass filters were used to isolate mass signals from measurement noise^8^, and subsequent median filtering (frame length of 49) and threshold detection were implemented such that all below-threshold points were set to zero and all above-threshold points were set to one. These filtered data provide a binary characterization of SMR occupancy seeing as the resonant frequency shifts caused by cell transit led to above-threshold measurements. Single-cell transit times were subsequently quantified by determining the number of consecutive above-threshold measurements collected for each cell.

### Code availability

Hardware control and analysis code is available from corresponding author S.R.M. upon reasonable request.

## Electronic supplementary material

Supplementary Information

Description of Additional Supplementary Files

Supplementary Movie 1

Supplementary Movie 2

Supplementary Movie 3

Supplementary Movie 4

Supplementary Movie 5

## Data Availability

Data supporting the findings of this study are available from the corresponding author upon reasonable request.
