# Supplementary material for: Microfluidic active loading of single cells enables analysis of complex clinical specimens
Source: Nat Commun. 2018 Nov 14;9:4784. doi: 10.1038/s41467-018-07283-x (PMC6235965; doi:10.1038/s41467-018-07283-x)
Supplement: Supplementary file 1 — Supplementary Information [file 41467_2018_7283_MOESM1_ESM.pdf]

Supplementary Information

Microfluidic active loading of single cells enables analysis of dilute and complex clinical samples

Nicholas L. Calistri, et al.

Type of detector	Measurement approach	Measurement time (ms)	Reference
**Electrical**			
	Impedance spectroscopy	60	[1] Cheung et al.
**Mechanical**			
	Optical stretching	1,000	[2] Guck et al.
	Solid constriction *(optical readout)*	100 to 1,000	[3] Rosenbluth et al.
	Solid constriction *(mass readout)*	100 to 1,000	[4] Byun et al.
	Hydrodynamic constriction	10	[5] Otto et al.
	Hydrodynamic stretching	0.1	[6] Gossett et al.
**Optical**			
	Image cytometry	10	[7] George et al.
	Image cytometry	100 to 1,000	[8] Wang et al.
	Raman spectroscopy	10,000	[9] Dochow et al.

Supplementary Table 1: Examples from the literature of measurement times for various types of microfluidic detectors.

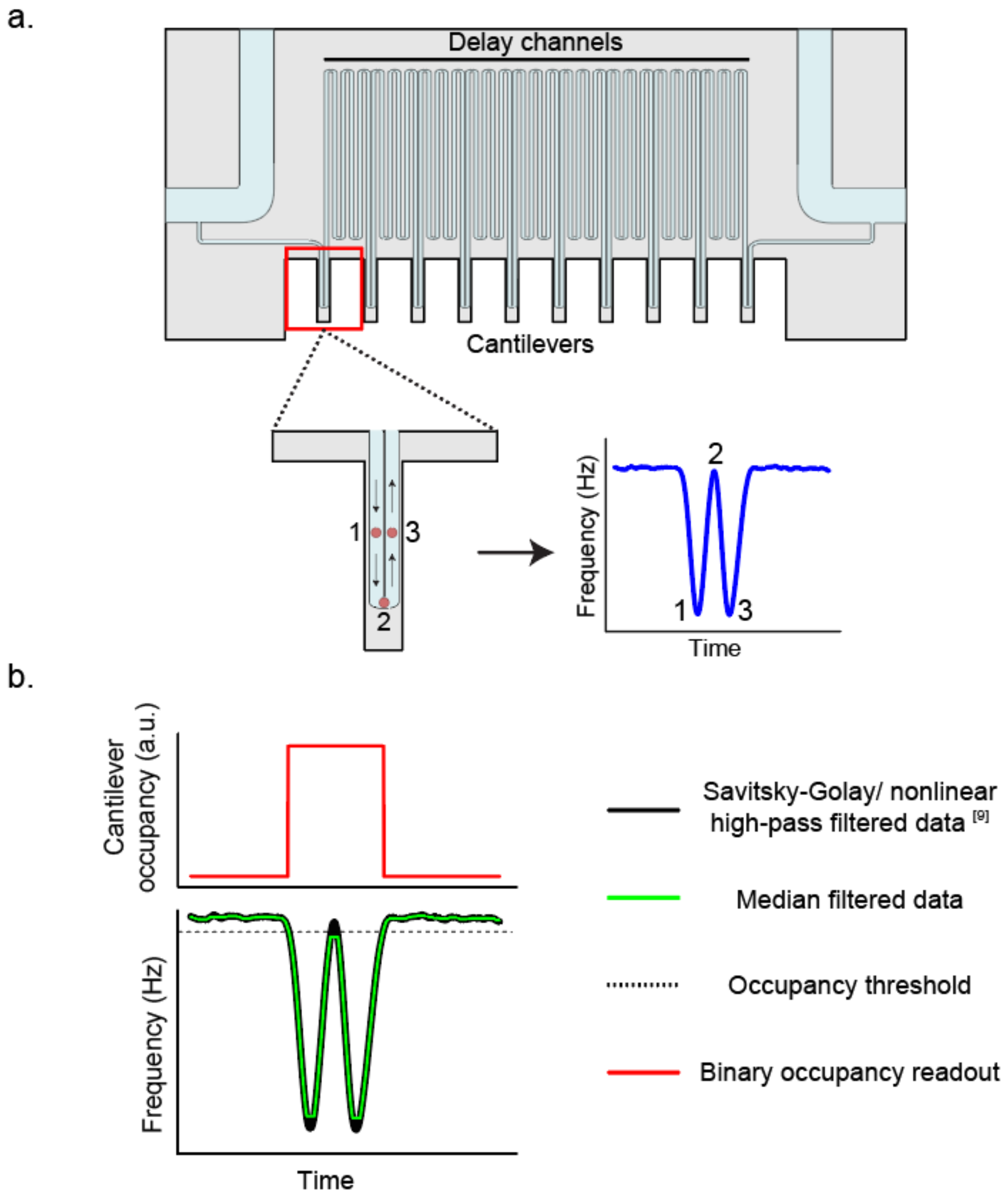

Supplementary Figure 1: Schematic of a serial suspended microchannel resonator (sSMR) platform **(a)**. The device consists of an array of SMR buoyant mass sensors placed periodically along the length of a long (50 cm) microfluidic measurement channel. The array is flanked on either side with two sampling channels with independent control of upstream and downstream pressures. For single-cell transit time measurements, the first cantilever of the sSMR was used to detect cell entrance in to the array (inset). The schematic of this cantilever demonstrates a cell flowing through the cantilever (left) and the corresponding resonant frequency measurements associated with these positions (right). **(b)** Representative plot showing the single-cell frequency measurements at various stages of filtering described in the **Methods**. The binary occupancy readout (red), shown here with the same time scale as the frequency data, indicates when the frequency shift is below the specified occupancy threshold (dashed line).

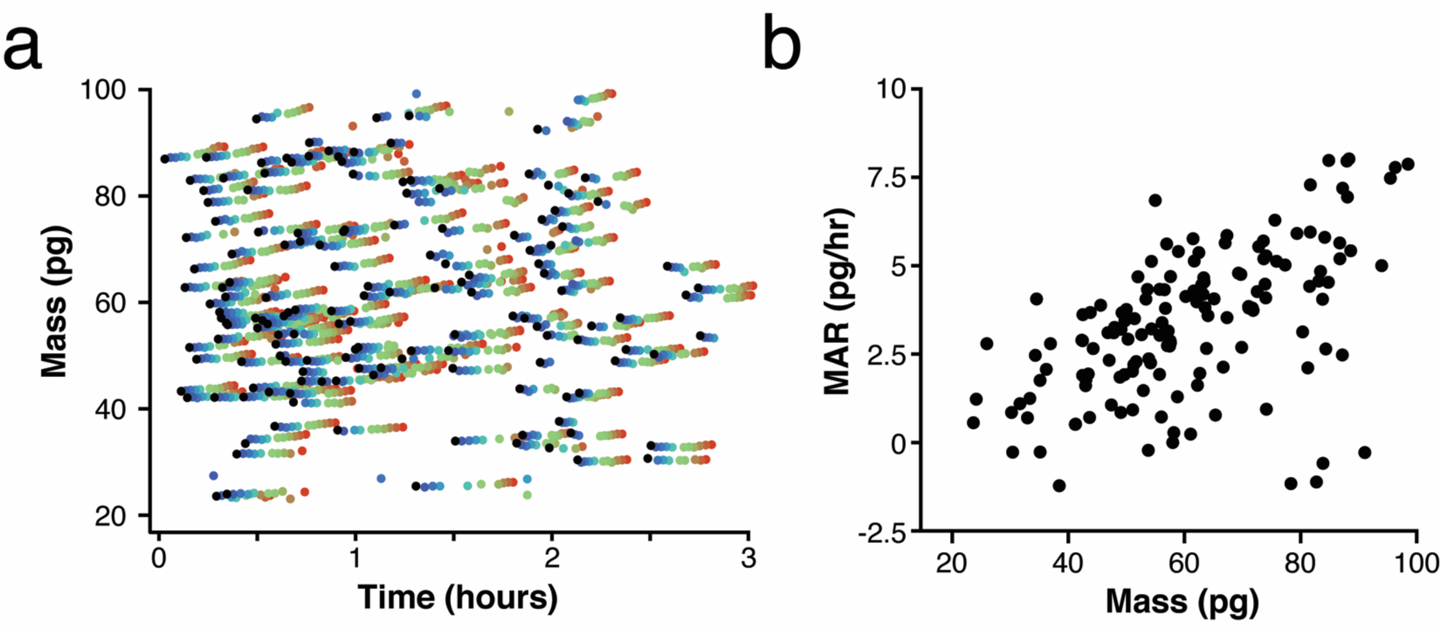

Supplementary Figure 2: Rare cell measurement of BaF3 cells **(a)** Dot plot of raw mass versus time data for BaF3 cells measured at each cantilever in a 12 cantilever sSMR device. Colored dots represent each individual cantilever, with the progression proceeding from black to blue to green to red as you move from the first to the last cantilever on the flow path. Single-cell trajectories are subjected to a linear fit to extract MAR. Cells were seeded by serial dilution at a density of 2.7×10^3 cells/mL, with ~270 total cells in 100 μL. 165 of the 270 cells (61%) were loaded into the array after 3 hours of measurement. **(b)** Dot plot of MAR versus mass for the same BaF3 cells.

Supplementary Note 1: Complete description of each function triggered by ROIs

State Name	Description
**[0] 'Loading flow'**	Sampling channel upstream and downstream pressures are equal
**[1] 'Queue forward'**	Sampling channel upstream pressure is slightly higher than downstream pressure but nodal pressure at measurement channel entrance remains the same as [0]
**[2] 'Queue backward'**	Same as [1], with reversed sampling flow direction (downstream pressure higher than upstream)
**[3] 'Major forward'**	Sampling channel upstream (cell sample reservoir) pressure is significantly higher than downstream pressure, but nodal pressure remains the same as [0]
**[4]'Major backward'**	Same as [3], with reversed sampling channel flow direction
**[5] 'Array kickback'**	Significant flow reversal in the measurement channel such that particles in the measurement array backflow towards the loading bypass
**[6] 'Array backflow'**	Minor flow reversal in the measurement channel
**[7] 'Seek forward'**	Sampling channel upstream pressure is moderately higher than downstream pressure, but nodal pressure remains the same as [0]
**[8] 'Seek backward'**	Same as [7], with reversed sampling channel flow direction

Supplementary Table 2: Pressure states involved in active loading

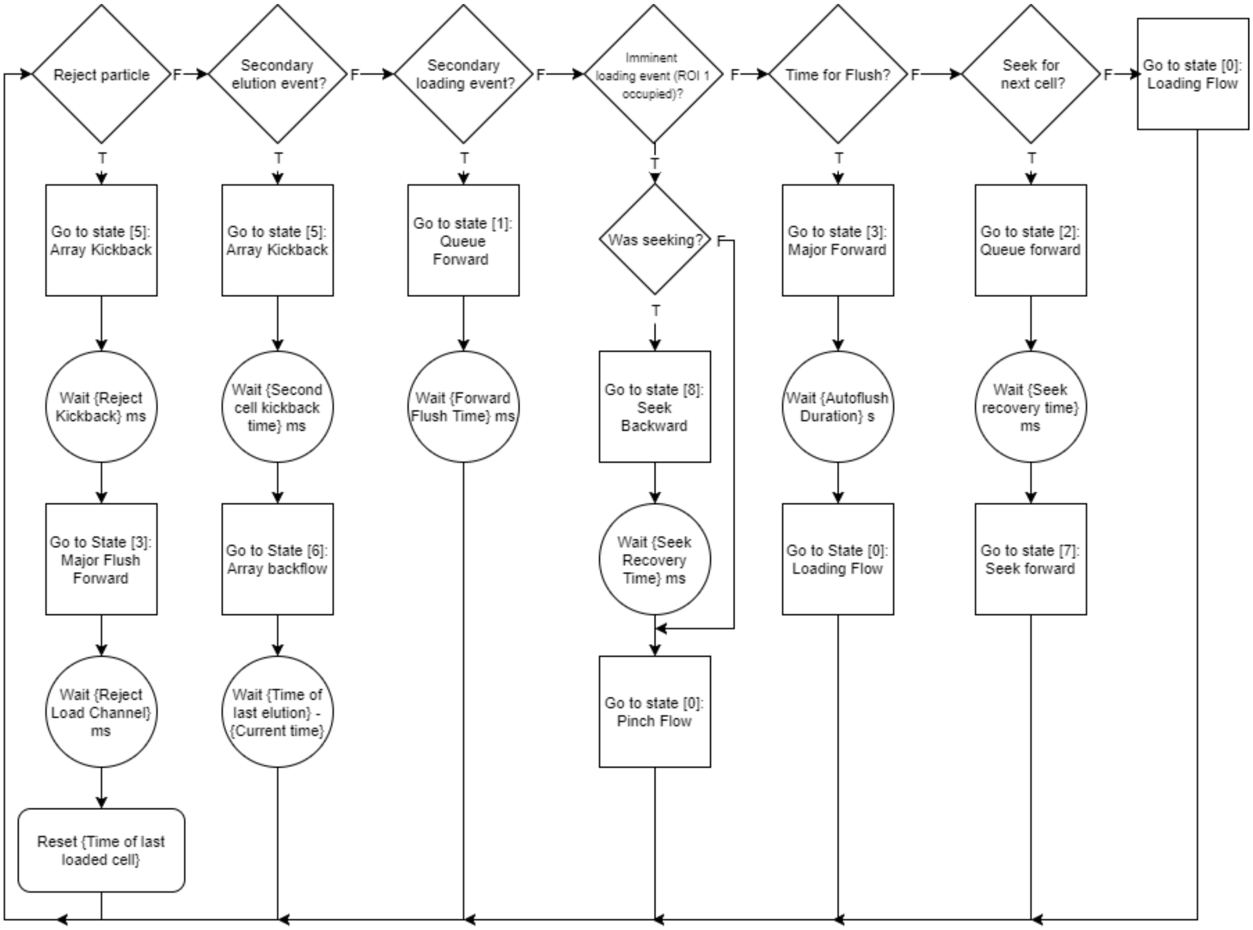

Supplementary Figure 3: Schematic of active loading code

Supplementary Note 2: Automated particle classification

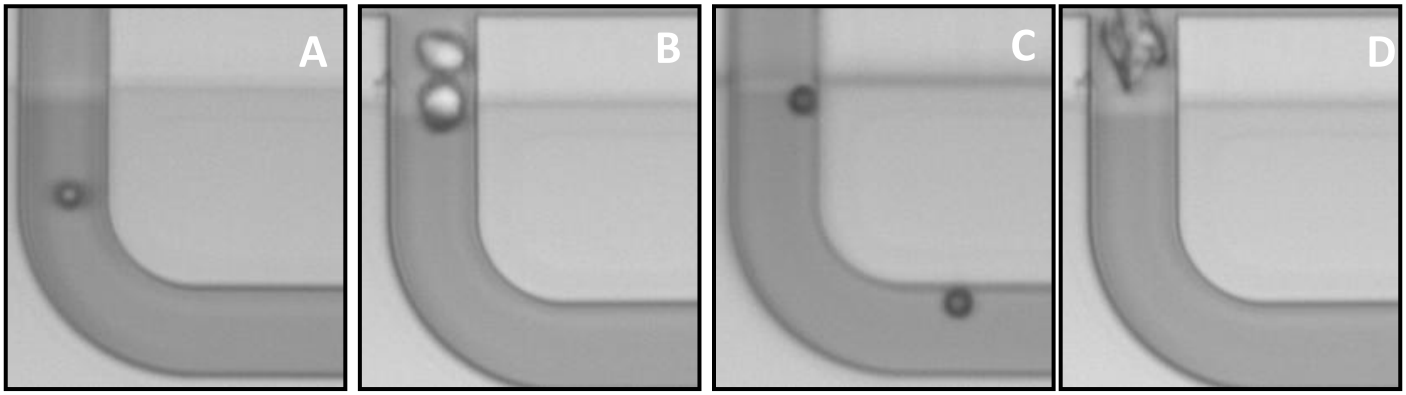

Supplementary Figure 4: Examples of particles automatically classified as a 'Singlet' (A), 'Doublet' (B), 'Multiple Singlet' (C), and 'Debris' (D).

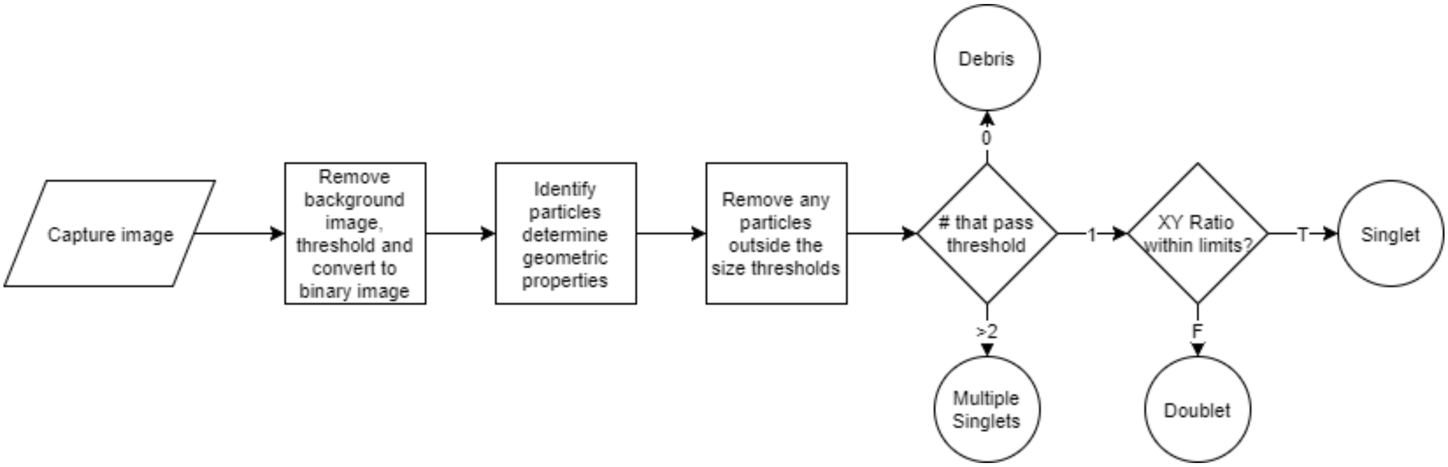

Supplementary Figure 5: Particle classification diagram depicting the automated particle classification logic. The background image is created by calculating the median value for each pixel from the past X frames, where X is a user designated control. The present frame is subtracted from the median image, effectively leaving behind an image showing only objects in motion. A user inputted pixel threshold is subtracted from the subtracted image, and the resultant values are coerced to a value between 0 and 255. The 'AutoBinaryThreshold' subVI is used to transform this image into a binary image, with pixel values of 0 or 1. Morphology of the resultant image is smoothed with automedian, dilate, convex hull, and hole filling subVIs. The 'Particle Analysis Report' subVI then identifies continuous pixel regions with a value of 1, and generates a list of these particles. Any particle outside of a user determined size (number of pixels) threshold is removed from the list. If there are no particles remaining, the triggering event is determined to have been 'Debris'. If there are more than one particles within the size threshold then the triggering event is determined to have been 'Multiple Singlets'. If only one particle is within the size threshold then the X:Y ratio of the bounding rectangle is used to determine whether the particle is a doublet. Particles with an X:Y ratio below the user designated threshold and above the reciprocal of the treshold are considered to be 'Singlets'. Particles with an X:Y ratio above the user designated threshold or below the reciprocol of the threshold are determined to be 'Doublets'.

Supplementary Note 3: Throughput enhancement provided by Active Loading

Here we present the throughput improvements that could be achieved by implementing active loading for various single-cell applications that have been described previously in the literature. For this purpose, we define the improvement metric as the ratio between the effective sampling flow rate and the flow rate that would have been achieved in the measurement channel without active loading. As we describe below, several assumptions are made in order to estimate the effects of detection and pneumatic control delay in the sampling channel and the ratio of cross sections of the measurement and sampling channels.

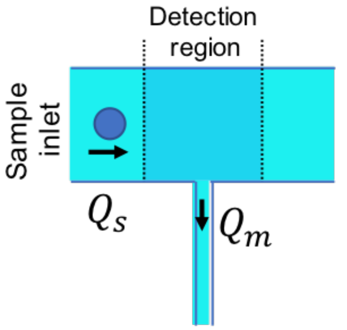

Supplementary Figure 6: Active loading detection region

Since each detection event during the 'seek' operation triggers a loading cycle (**Supplementary Video 1**), the throughput with active loading is a function of cell concentration in the sample. Within the non-zero time frame of the loading cycle, the seeking flow is stopped, reducing the effective sampling flow rate (Q_t). We define the effective flow rate as:

$$Q_t = \frac{V}{T_t} \tag{1}$$

where V and T_t are the total sample volume to be measured and total duration of sampling, respectively. Assuming a time frame of t_L is required to load each cell in to the measurement channel from the moment of detection, we can calculate the total measurement duration (T_t) as a function of cell concentration (C) as follows:

$$T_t = T_s + CVt_L \tag{2}$$

where T_s is the total time required to flow the same sample of volume V at a flow rate of Q_s with no particle-detection. Inserting Equation (2) into (1), we get

$$Q_t = \frac{V}{(T_s + CVt_L)} = \frac{1}{\frac{1}{Q_s} + t_L C} \tag{3}$$

This is a general equation defining the effective flow rate provided by active loading, when the detection and loading events are taken into account. We model the time required to load each cell into the measurement channel assuming non-ideal system components with non-zero time responses. In the figure below, we illustrate the change of flow rate in the sampling channel as a function of time during a cell loading cycle. The loading cycle starts when a cell is detected in the sampling channel as it is flowing at a seeking flowrate of Q_s. The latency due to the pneumatic instrumentation and the detection scheme cause a detected cell to miss the entrance of the measurement channel, creating an excess volume (shaded) to be sampled into the measurement channel before

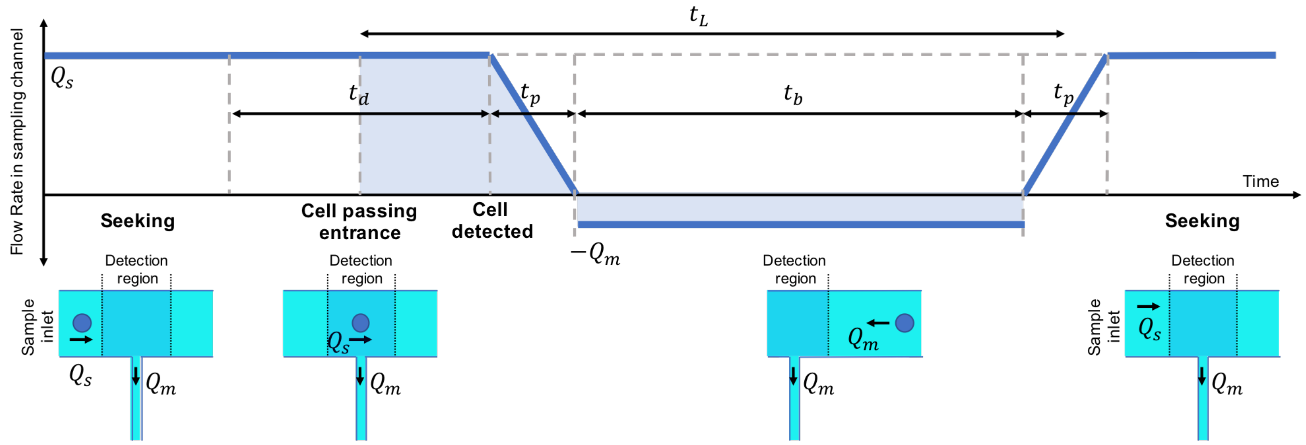

Supplementary Figure 7: Timeline of Active Loading events

the detected particle. For simplicity, we define two fundamental time delays dictated by the detection time (t_d) and pneumatic latency (t_p). We assume that before the cell enters the measurement channel, all excess volume is loaded into the measurement channel at a flow rate of Q_m, which determines the time required to back flow a cell into the measurement channel (t_b). Since the sampling into the measurement channel is from downstream only, the detection region is centered at the channel entrance, and the pneumatic response is linear in time, we can approximate the loading time of a detected cell as:

$$t_L = \frac{t_d}{2} + \frac{3t_p}{2} + \left(\frac{t_d}{2} + \frac{t_p}{2}\right)\frac{Q_s}{Q_m} = \frac{t_d + 3t_p}{2} + \frac{(t_d + t_p)Q_s}{2Q_m} \tag{4}$$

Here Q_m is the flow rate in the measurement channel and inversely proportional to the measurement time required for the targeted application (or proportional to the measurement bandwidth) and kept constant at all times during the seeking and loading cycles. For the purpose of this analysis, we assumed that the backflow rate is identical to the measurement flow rate. However, faster rates could be utilized with more complicated control algorithms, which would require the replacement of Q_m in Equation (4) above. As the merit of active loading relies on achieving $Q_s \gg Q_m$, Equation (4) simplifies to

$$t_L \approx \frac{(t_d + t_p)Q_s}{2Q_m} \tag{5}$$

Using Equations (3) and (5), we calculate the net improvement of active loading as a function of system and sample variables as:

$$\frac{Q_t}{Q_m} = \frac{1}{\frac{Q_m}{Q_s} + \frac{(t_d + t_p)CQ_s}{2}} \tag{6}$$

Equation (6) shows that the throughput improvement for a given sample concentration is a function of the seeking flow rate. Due to the non-zero time response of the detector and pneumatics, the seeking flow rate has an optimal value to achieve the maximum throughput improvement for a given cell concentration. We calculate this optimal rate (Q_s') as a function of system variables, sample concentration and measurement flow rate requirement by taking the derivative of Equation (6), equating it to zero and solving for Q_s:

$$Q_s' = \sqrt{\frac{2Q_m}{(t_d + t_p)C}} \tag{7}$$

Finally, we calculate the throughput improvement from active loading at the optimal seeking flow rate by inserting Equation (7) into (6):

$$\left.\frac{Q_t}{Q_m}\right|_{Q_s = Q_s'} = \frac{1}{\sqrt{2(t_d + t_p)CQ_m}} \tag{8}$$

Equation (8) demonstrates that the benefit of active loading increases for samples that are low in concentration, for applications where a slow measurement flow rate is necessary and for measurement systems with low latency.

In the equations above, t_d is defined by the method utilized for detecting cells in the sampling channel. Although faster detection methods such as electrical, capacitive, interferometric could be utilized here, we will focus on detection by imaging as it provides additional benefits for active loading, e.g. debris rejection, cell shape determination, fluorescence measurements etc. For the special case of the optical detection using a camera, we will conservatively assume that 4 frames are necessary to successfully detect a cell at the shutter speed of the camera, setting $t_d = 4/f_r$. Therefore, the frame rate and field of view puts an upper bound on Q_s. Using Equations (6-8), we plot below the throughput improvement for a range of sample concentrations for the system used in this

paper (Current System) and for a system with the same channel dimensions but faster detection and pneumatic control (Fast System). For these two scenarios, we use the specifications listed in the adjacent table. We assume the size of the detection region to be centered around the measurement channel entrance and 200 microns long. Therefore, a camera that has a faster shutter speed would enable faster seeking flow rates, increasing the throughput improvement for samples with low concentration of cells.

The plot below shows that the throughput improvement is a strong function of sample concentration and that a more than 100-fold improvement is theoretically possible for low concentration samples. Although the benefit of active loading drops for samples that are concentrated, fast pneumatics and detection schemes could still enable a more than 10-fold improvement over traditional methods.

	Current system	Fast system*
f_r	150 fps	1000 fps
t_d	~27 ms	4 ms
ROI 1 length	200 μm	200 μm
t_p	25 ms	5 ms

*Dolemite #3200531 camera and Bibus 860 series solenoid

Supplementary Table 3: Specifications of current system and proposed 'Fast' system.

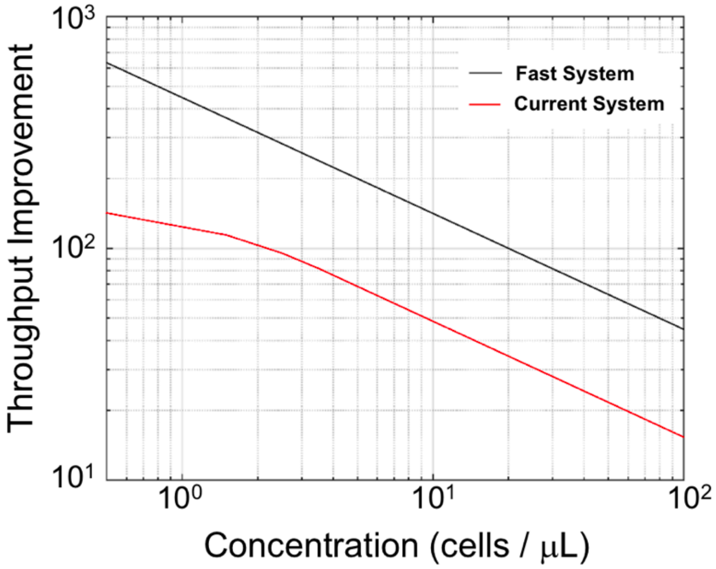

Supplementary Figure 8: Throughput improvement at different concentrations

Finally, we determined the extent to which other single-cell microfluidic sensors could benefit from the active loading approach. In the table below, we estimate theoretical throughput improvements possible with active loading if applied to various single-cell measurement techniques. For conducting a fair comparison, we assumed the same flow speed that was used in the corresponding reference is achieved in the measurement channel we utilized in this work. Then we calculated the optimal seeking flow rate for our current system and a fast system. In the event that the optimal seeking flow rate exceeded what is achievable with the sampling channel camera we instead used the maximum achievable flow rate.

Method	Time (ms)	Flow rate (μL/h)	1 cell /μL		10 cells / μL		100 cells / μL	
			Current	*Fast*	*Current*	*Fast*	*Current*	*Fast*
Cheung et al.[1]	60	29	**6**	**36**	**5**	**24**	**3**	**8**
Guck et al.[2]	1000	5	**8**	**24**	**3**	**8**	**1**	**2**
Rosenbluth et al. [3]	100-1000	36	**5**	**28**	**4**	**16**	**2**	**5**
Byun et al.[4]	100-1000	4	**40**	**235**	**32**	**112**	**12**	**35**
Otto et al.[5]	10	144	**1**	**8**	**1**	**7**	**1**	**5**
Wang et al.[8]	1000	0.3	**216**	**630**	**68**	**200**	**21**	**63**

Supplementary Table 4: Throughput improvement (numbers in bold) for applying active loading to previously published single-cell measurements. Throughput improvement is defined by the ratio between the effective sampling flow rate and the flow rate that would have been achieved in the measurement channel without active loading. A value of unity indicates that there would be no improvement from active loading.

Supplementary Note 4: Throughput modeling with desired minimum particle spacing

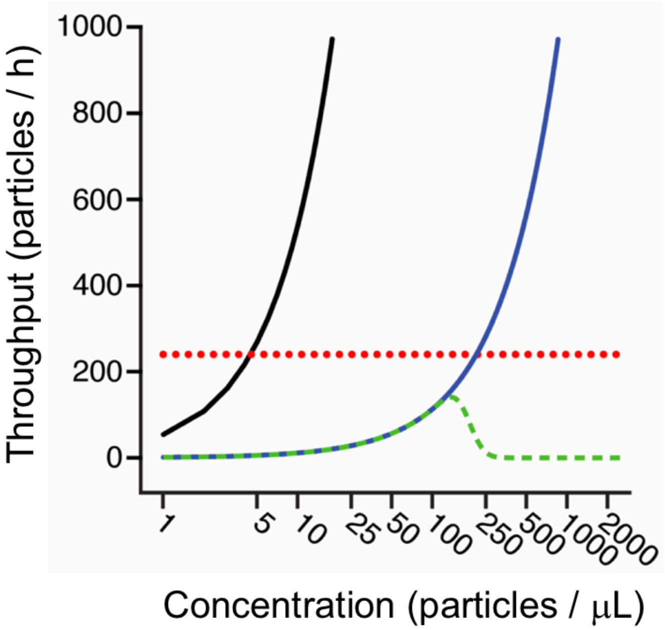

Supplementary Figure 9: Throughput of the current system at different concentrations

The throughput achievable by passively loading cells into a sSMR chip is Poisson limited. The average throughput ($F_{Passive}$) is equal to the concentration (C) of cells in the sample multiplied by the volumetric flow rate (Q_V) through the chip, where V is the total chip volume and T is the total time required for a cell to travel through the entire chip:

$$F_{Passive} = CQ_V = C\frac{V}{T} \tag{9}$$

As previously shown by Cermak *et al.*[9], the precision of mass accumulation rate measurements made by a sSMR array is inversely proportional to T. Therefore, to achieve a biologically relevant measurement precision, we keep the volumetric flow rate through the sSMR chip constant such that, on average, cells travel through the chip in ~15 minutes. A constant volumetric flow rate (Q_V) in (9) results in a concentration-limited throughput. Our sSMR devices for mammalian cells have a volume of 0.283 µL, resulting in a volumetric flow rate of approximately 1.132 µL/h. For this case, Equation (9) simplifies to:

$$F_{Passive} = 1.132\ \mu\text{L/h}\ \times C \tag{10}$$

which is plotted with a blue line in the plot above.

Equation (10) represents an idealized case where all of the cells flow at an identical velocity in the measurement channel. Since measuring MAR of a cell requires a set of mass measurements performed by different sensors in the sSMR chip to be assigned to the same cell, variations of cell order in the measurement channel could create discrepancies during this matching process [9]. Cells or particles in the measurement channel have varied velocities that depend on their size and position in the channel. Interaction of cells with channel walls exacerbates this problem by slowing certain cells in the stream. Furthermore, doublet formation in the measurement channel, or from simultaneously loading collisions in high concentration samples, results in clogging. To address these limitations, we empirically determined a minimum time gap of 15 seconds between events to prevent most collisions and changes in cell order. The average time difference between each cell loading event, $\overline{t_\Delta}$ can be calculated by:

$$\overline{t_\Delta} = \frac{1}{F_{Passive}} \tag{11}$$

A Poisson probability distribution for time between each loading event can be calculated using Equation (11), which is used to find the fraction of events with a greater-than 15 second spacing for any given concentration.

$$P(t \geq 15s) = 1 - \frac{e^{-\lambda}\lambda^k}{k!} = 1 - \frac{(e^{-\overline{t_\Delta}})(\overline{t_\Delta}\ ^t)}{t!} \tag{12}$$

The effective rate of particles (F_{eff}) is defined as the rate of particles with a time gap of at least 15 seconds between the leading and trailing particle. F_{eff} is thus calculated as the rate of particles entering the array multiplied by the probability of a time gap greater than 15 seconds squared (green dashed line in plot above):

$$F_{eff} = (C \times 1.132\ \mu\text{L/h}) \times P(t \geq 15s)^2 \tag{13}$$

The maximum theoretical active loading throughput would be achieved with instantaneous detection and loading from the sampling channel. The maximum throughput would then be divided into a 'seek' limited fraction and a 'queue' limited fraction. The seek limited throughput limit can be calculated by using Equation (9) and substituting the seeking volumetric rate for the device volumetric rate. (plotted as the black line in the plot above):

$$F_{active} = 54\ \mu\text{L/h} \times C \tag{14}$$

To calculate this 'queue' limited portion of active loading, we assumed a time delay of 15 seconds that minimizes matching failure, as previously described. The throughput in this case is simply calculated by assuming a uniform loading every 15 seconds (dotted red line in plot above):

$$F_{active} = \frac{1}{t_{gap}} = \frac{1}{15}\text{cells/s} = 240\ \text{cells/h} \tag{15}$$

The theoretical throughput curve presented in **Fig. 2c** is constructed by taking the minimum throughput of either the 'seek' or 'queue' limited conditions for a particular concentration. As seen in **Fig. 2**, the experimental throughput of the sSMR achieved with active loading does not match this theoretical maximum, particularly for low-concentration samples. This discrepancy is due to the practical throughout limitations imposed by the system's optical and pneumatic components described in **Supplementary Note 3.**

Supplementary Note 5: Accuracy of the real-time cell classification used for active loading

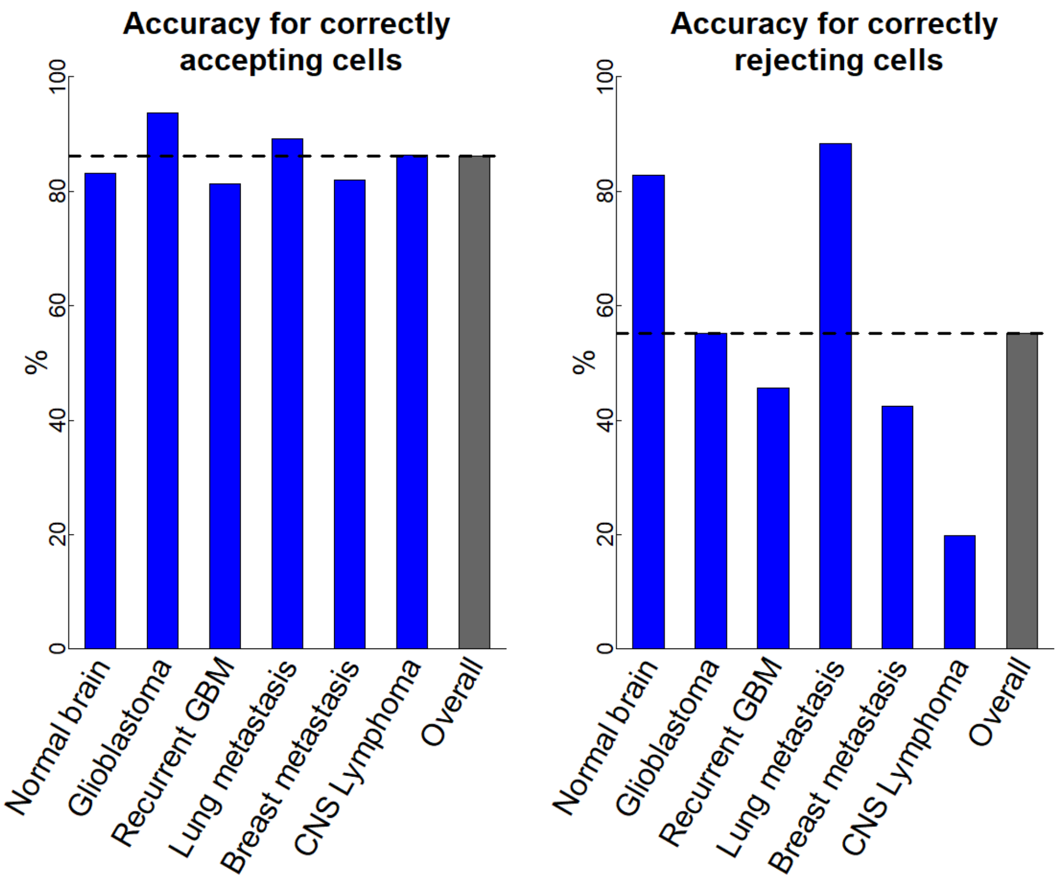

Supplementary Figure 10: Real-time particle classification accuracy

Here we evaluate the accuracy of active loading for correctly allowing cells into the measurement channel based on user-specified criteria of the brightfield images that are acquired as cells transition from the sampling channel into the measurement channel. Each image was analyzed in real-time by Labview code in order to assess whether or not the particle should be allowed into the measurement channel (accepted) or removed via the sampling channel (rejected). After the experiment, each image was evaluated manually to determine if the real-time decision based on the automated image analysis was correct. User-specified criteria were designed to reject particles that were classified as 'Doublet', 'Multiple Singlets', or 'Debris'. When combining all six samples together, the accuracy for correctly allowing particles into the measurement channel was 86% (2040 particles were allowed by the real-time code, 1757 of them were manually classified as single cells) and 55% for correctly rejecting particles (4159 particles rejected by the real-time code, 2295 of them were manually classified as rejection events). The accuracy for each sample is shown in the figure above where we plot the percentage of real-time classifications that are in agreement with the manual validation.

For this application, user-specified settings are typically weighted to avoid rejection criteria. Consequently, this approach tolerates higher rates of single-cell rejection, despite the fact these events should have been accepted. Rejection of single cells is not particularly detrimental to throughput because the seeking code is capable of quickly finding a second event to load into the array, and lowers the probability that debris or clumps of cells may interfere with flow in the measurement channel. Furthermore, the rejected events are recovered in the downstream collection tube, and for situations were sample is limited, the tube could be reloaded back into the system. In some cases, vibration of the instrument from nearby disturbances triggered the acquisition

of an image that did not contain a particle. These events, which were not detrimental to the experiment, were not included in our accuracy assessment.

<u>Supplementary Note 6:</u> Cost of goods for active loading

Item Number	Short name	Catalog name	Cat #	Seller	Quantity needed	Price per unit	Total price
*1	Regulator	Electronic Pressure regulators	QPV1TFEE030CXL	Proportion Air	3	$535.00	$ 1,605.00
*2	Regulator cord	Proportionair powercord 10'	QBT-C-10	Proportion Air	3	$35.50	$ 106.50
*3	Camera	Monochrome USB 3 Camera	BFS-U3-13Y3M-C	FLIR	1	$395.00	$ 395.00
*4	Camera Cord	USB 3.1 Type-A to Micro-B (Locking) Cable	ACC-01-2300	FLIR	1	$10.00	$ 10.00
5	PEEK	.005" ID PEEK tubing (5 ft)	1576	IDEX H&S	1	$49.38	$ 49.38
6	Tygon	Clear tubing OD 1/8" ID 1/16" (50ft)	C210A-0102	Grainger	1	$38.00	$ 38.00
7	1/4" OD Pneumatic tubing	Flame-Retardant Polyethylene Opaque Tubing for Air	5156K87	McMaster Carr	25	$0.36	$ 9.00
8	Manual regulator	0-60psi Manual Regulator	PRG101-60	Omega	1	$300.00	$ 300.00
9	DAQ	±10 V, Analog Output, 100 kS/s, 4 Ch Module	779012-01 (NI-9263)	National Instruments	1	$416.00	$ 416.00
10	DAQ Chassis	C Series USB Single Module Carrier	781425-01 (NI-USB 9171)	National Instruments	1	$287.00	$ 287.00
11	Wheaton Vials	Wheaton sample vials - white top 20mL (case of 72)	80076-578	VWR	1	$112.00	$ 112.00
12	Regulator power supply	24 V 24W AC/DC Wall mount adapter	62-1246-ND	DigiKey	1	$20.00	$ 20.00
13	1/8" NPT to 1/4" OD Push to connect	1/8" NPT to 1/4" OD Push to connect adapters	5779K102	McMaster Carr	6	$2.96	$ 17.76
14	Microscope	Meiji VM-2V Vertical Mount	VM-2v w/ FL20	Meiji	1	$1,477.00	$ 1,477.00
15	Microscope Stand	Meiji Stand Pole type stand /w coarse & fine adjustment	VM-PC-Stand	Meiji	1	$660.00	$ 660.00
16	Objective	S. Plan Objective, 10x	MA337	Meiji	1	$150.00	$ 150.00
17	Light source	Mounted LED and driver	LEDD1B, MNWHL4	Thorlabs	1	$449.00	$ 449.00
18	Illumination adapter	Lasercut acryllic mounting adapter	Custom	Custom	1	$50.00	$ 50.00
						**Total cost:**	**$ 6,101.64**

Supplementary Table 5: The cost of goods sheet for constructing a system from scratch that is capable of performing the active loading tasks in a simple microfluidic H channel. The starred items (*) represent items required to upgrade an existing sSMR system to be compatible with the active loading method. The system requires the additional purchase of one each of these items for a total upgrade cost of $975.50.

Supplementary Note 7: Primary sample handling

The six primary samples underwent the same protocol with regards to disassociation, recovery, and drugging however the exact timeline of each tissue varied slightly based on the amount of tissue and drug used. After at least [culture time] in culture (with the exception of CNS lymphoma which was cultured for 24 hours), persistent red blood cells were removed with RBC lysis buffer (00-433-57, Thermo Fisher Scientific). The remaining cells were then dissociated with Accutase (A6964, Sigma-Aldrich) and further purified via demyelination (130-096-733, Miltenyi Biotec) with MS separation columns (130-042-201, Miltenyi Biotec), or debris removal (130-109-398, Miltenyi Biotec). The purified cells were plated in 6 or 24 well plates and allowed to recover in the well plate for [recovery time] before addition of the drug. After [drug duration] days, the samples were prepared for sSMR for drug response measurements by dissociation into a single-cell suspension using Accutase and gentle pipetting. Cells were resuspended at a concentration of 100,000 cells/mL in Neurocult NS-A (as prepared above) with the same concentration of drug or DMSO as their respective culture.

Culture Timeline

Tissue Type	[culture time] (days)	[recovery time] (days)	[drug duration] (days)	Vehicle	Drug 1	Drug 2
**Normal brain**	2	2	3	DMSO	250 µM TMZ	
**Glioblastoma**	5	5	8	DMSO	250 µM TMZ	
**Recurrent Glioblastoma**	2	4	3	DMSO	1 µM Abema	
**Breast Met**	3	4	3	DMSO	1 nM RAD	100 nM Abema
**Lung Met**	3	5	3	Water	100 µM Carbo	
**CNS Lymphoma**	1	1	2	DMSO	10 nM Ibrutinib	

Supplementary Table 6: Culture timeline for primary samples

Sample Viability

Tissue Type	Vehicle Viability (Live/Dead)	Drug 1 Viability (Live/Dead)	Drug 2 Viability (Live/Dead)
**Normal brain**	35%	30%	
**Glioblastoma**	73%	69%	
**Recurrent Glioblastoma**	67%	59%	
**Breast Met**	35%	32%	35%
**Lung Met**	80%	74%	
**CNS Lymphoma**	74%	65%	

Supplementary Table 7: Primary sample viabilities

Supplementary Note 8: Primary sample biomarkers and pathology

Primary Tissue Type	Drug Assessed	Notes
**Normal Brain**	Temozolomide	Normal brain was used as a negative control for drug response as well as baseline mass accumulation, due to its lack *in vitro* cell replication.
**Glioblastoma**	Temozolomide	Temozolomide is part of the standard of care treatment for glioblastoma[14].<br>Molecular analysis on this sample showed unmethylated MGMT status, a biomarker associated with resistance to temozolomide.
**Recurrent Glioblastoma**	Abemaciclib	Abemaciclib is currently being tested in clinical trials of newly-diagnosed and recurrent glioblastoma[15]. In tumor cells, RB1 mutation/deletion is a known resistance mechanism to abemaciclib.<br>Biomarker analyses did not show RB1 alteration in this tumor sample.
**Breast adenocarcinoma metastasis**	Abemaciclib	Abemaciclib is a US Food and Drug Administration (FDA)-approved therapy for the treatment of hormone receptor (HR)-positive, human epidermal growth factor receptor 2 (HER2)-negative advanced or metastatic breast cancer[16].<br>Pathological analysis of this sample showed HR-positive and HER2-negative statuses.
	RAD001	RAD001 (everolimus) is another FDA-approved therapy for the treatment of hormone receptor (HR)-positive, human epidermal growth factor receptor 2 (HER2)-negative advanced or metastatic breast cancer[16].
**Non–small cell lung cancer (NSCLC) metastasis**	Carboplatin	Carboplatin is part of the standard of care for the treatment of metastatic NSCLC without activating EGFR, ROS1, ALK or BRAF mutation[17].<br>Histomolecular analyses of this sample showed absence of EGFR, ROS1, ALK or BRAF mutation.
**Primary CNS Lymphoma**	Ibrutinib	Ibrutinib is an FDA-approved therapy for the treatment of several subtypes of lymphoma, and is currently evaluated in primary CNS lymphoma within clinical trials[18].

Supplementary Table 8: Primary sample therapy information

<u>Supplementary Note 9:</u> BT1417 - Normal brain information

	Buoyant Mass	MAR	MAR per Mass
**DMSO-TMZ p-value**	0.713	0.849	0.837

Supplementary Table 9: BT1417 biophysical measurement significance

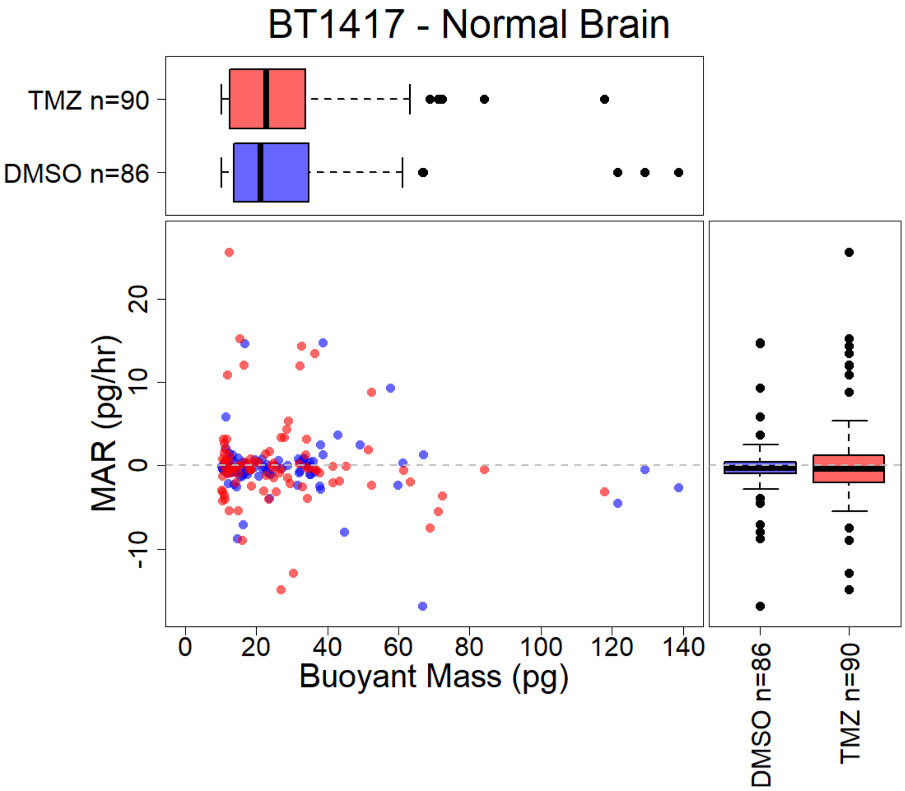

Supplementary Figure 11: Biophysical measurements of BT1417

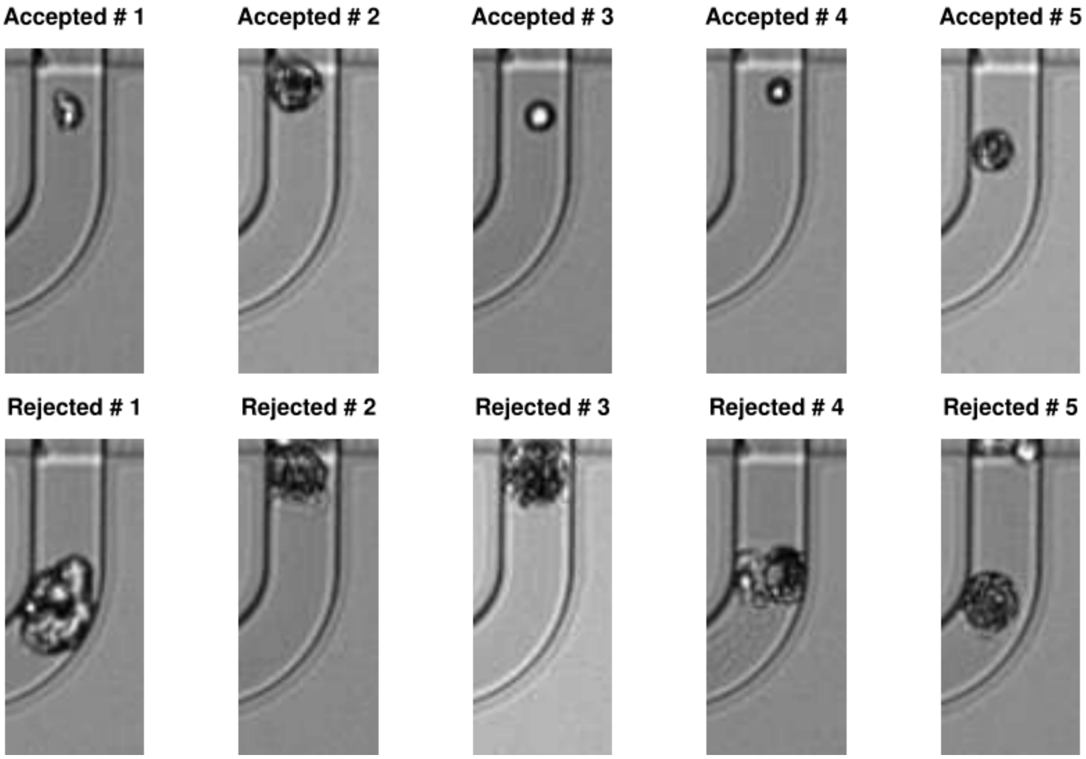

Supplementary Figure 12: Representative images of accepted/rejected cells

Supplementary Note 10: BT1410 – Glioblastoma information

	Buoyant Mass	MAR	MAR per Mass
**DMSO-TMZ p-value**	0.0517	0.937	0.545

Supplementary Table 10: BT1410 biophysical measurement significance

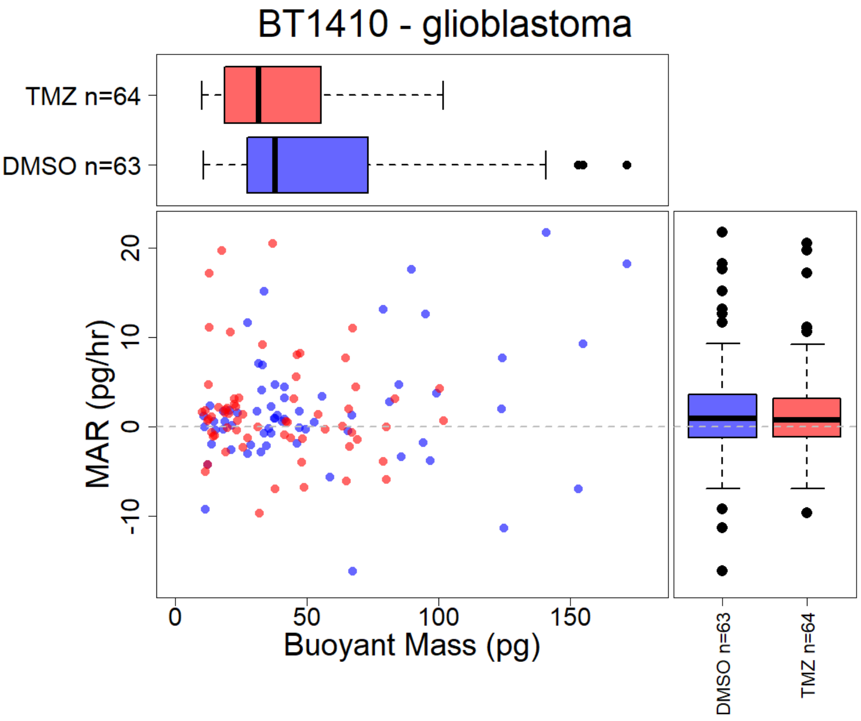

Supplementary Figure 13: Biophysical measurements of BT1410

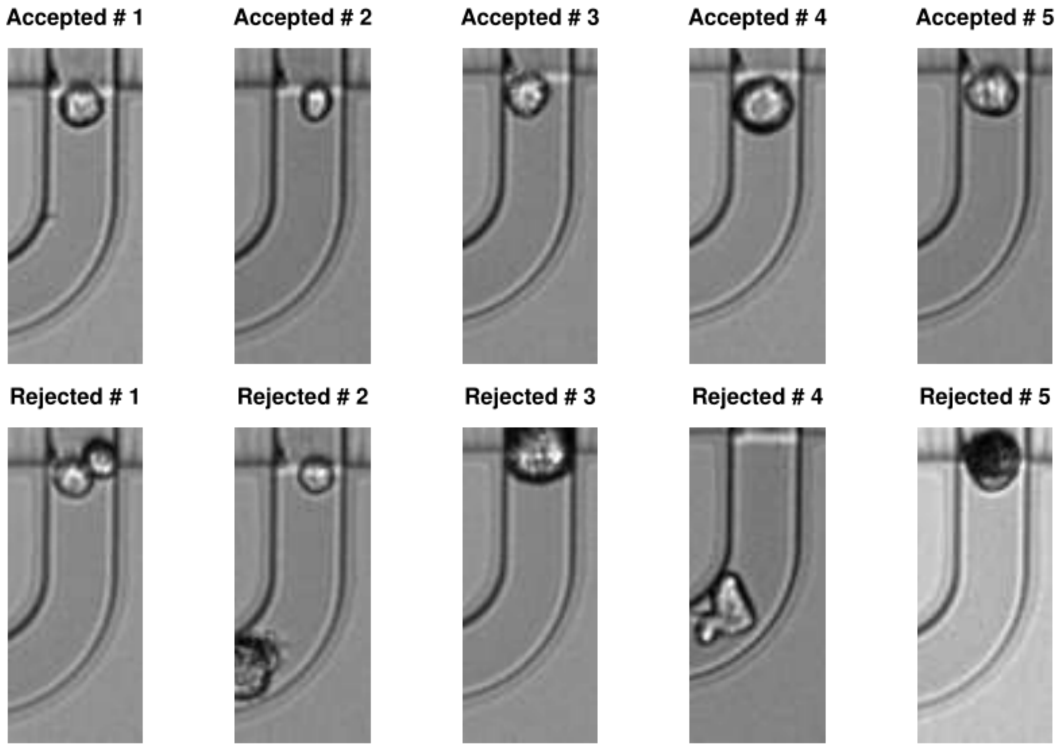

Supplementary Figure 14: Representative images of accepted/rejected cells

Supplementary Note 11: BT1233 – Recurrent glioblastoma information

	Buoyant Mass	MAR	MAR per Mass
**DMSO-Abemaciclib p-value**	0.164	0.0298	0.032

Supplementary Table 11: BT1233 biophysical measurement significance

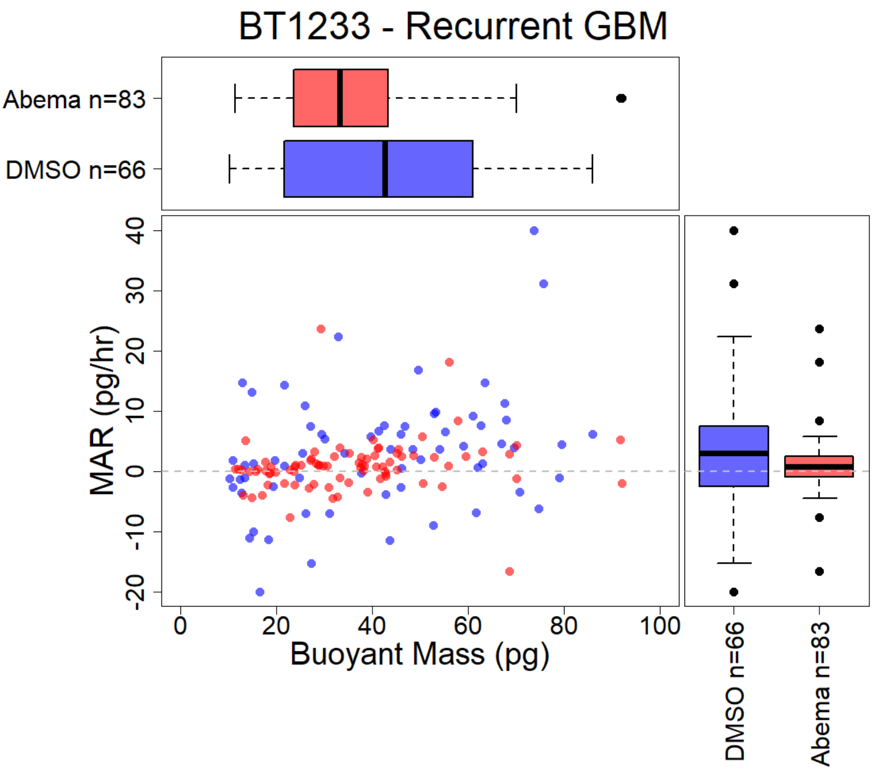

Supplementary Figure 15: Biophysical measurements of BT1233

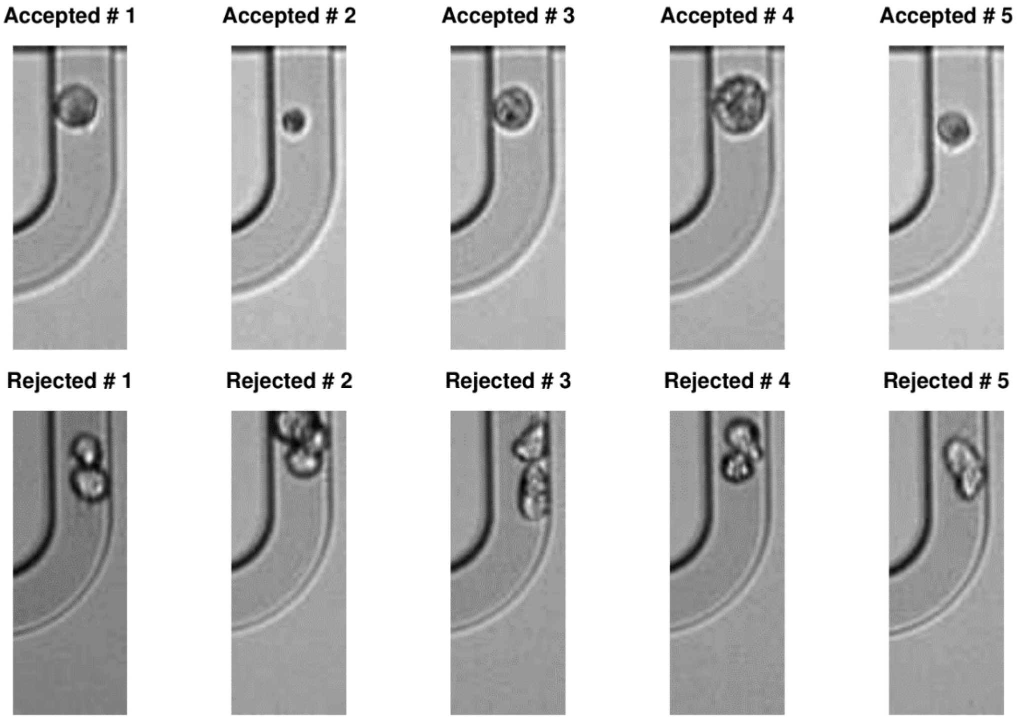

Supplementary Figure 16: Representative images of accepted/rejected cells

Supplementary Note 12: BT1419 – Breast metastasis information

	Buoyant Mass	MAR	MAR per Mass
**DMSO-RAD001 p-value**	0.264	0.966	0.916
**DMSO-Abemaciclib p-value**	0.744	0.0240	0.0290

Supplementary Table 12: BT1419 biophysical measurement significance

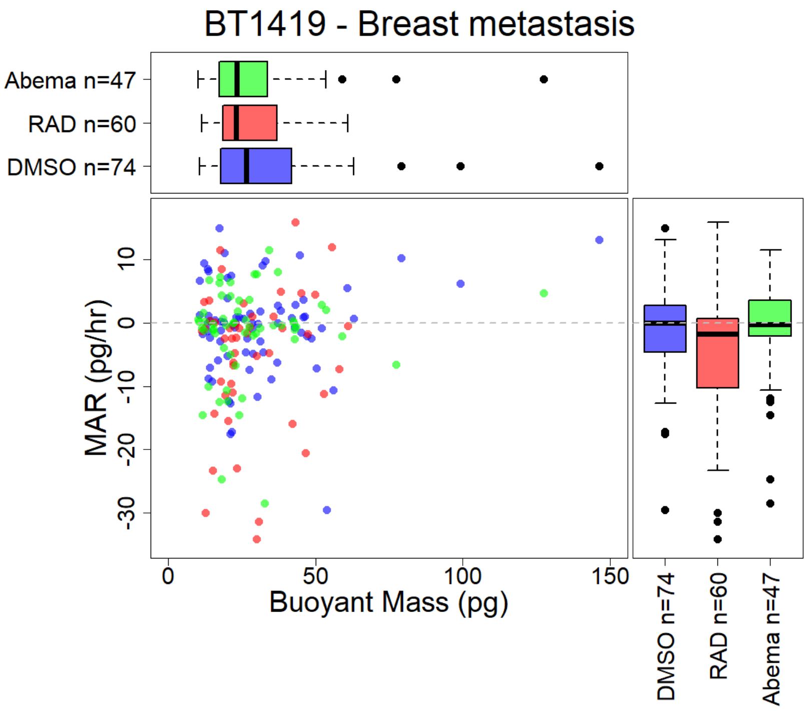

Supplementary Figure 17: Biophysical measurements of BT1419

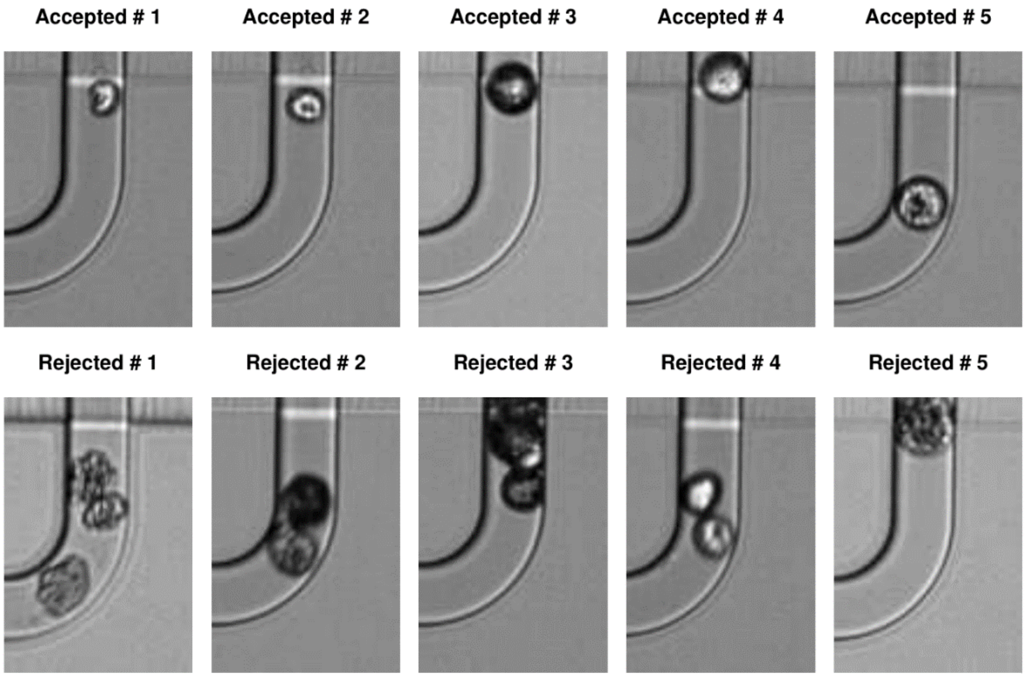

Supplementary Figure 18: Representative images of accepted/rejected cells

Supplementary Note 13: BT1443 – Lung metastasis information

	Buoyant Mass	MAR	MAR per Mass
**DMSO-Carboplatin p-value**	0.998	0.0931	0.0251

Supplementary Table 13: BT1443 biophysical measurement significance

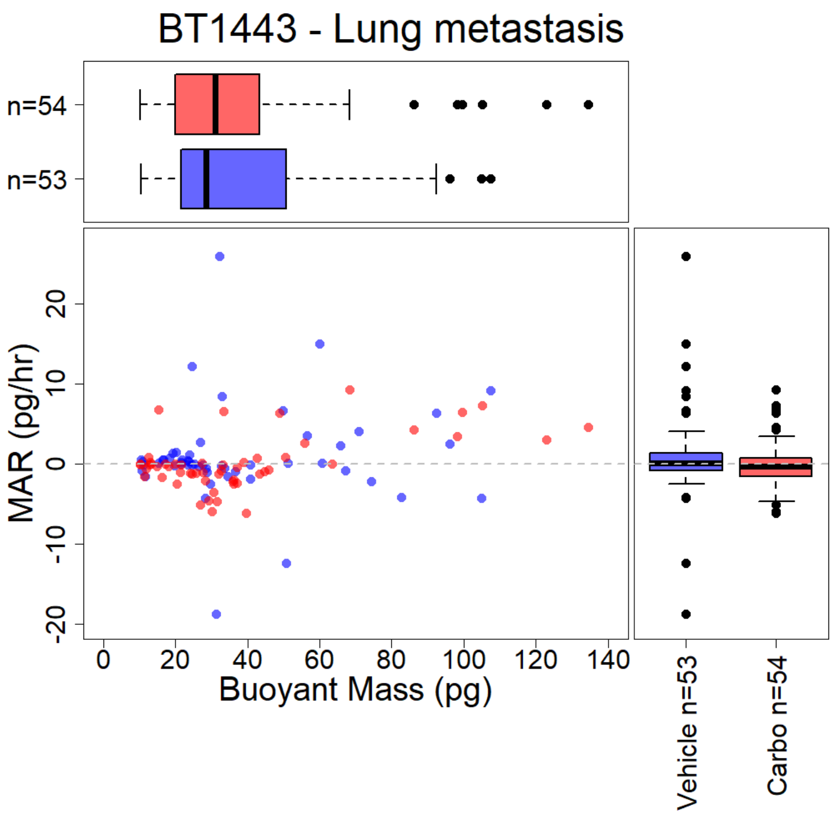

Supplementary Figure 19: Biophysical measurements of BT1443

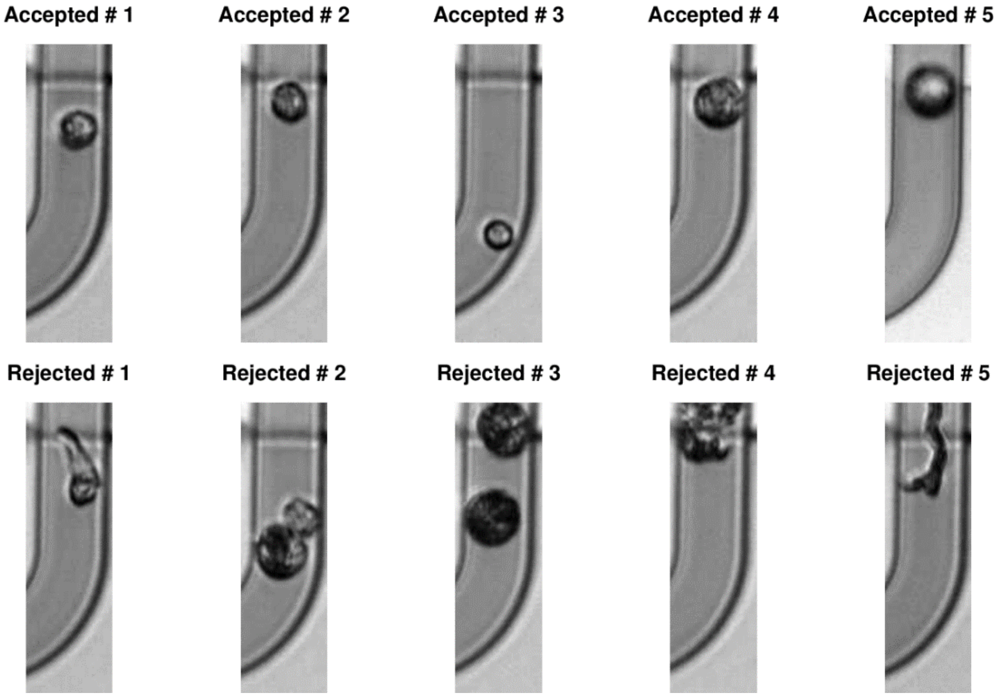

Supplementary Figure 20: Representative images of accepted/rejected cells

Supplementary Note 14: BT1448 – CNS lymphoma information

	Buoyant Mass	MAR	MAR per Mass
**DMSO-Ibrutinib p-value**	0.600	0.184	0.203

Supplementary Table 14: BT1448 biophysical measurement significance

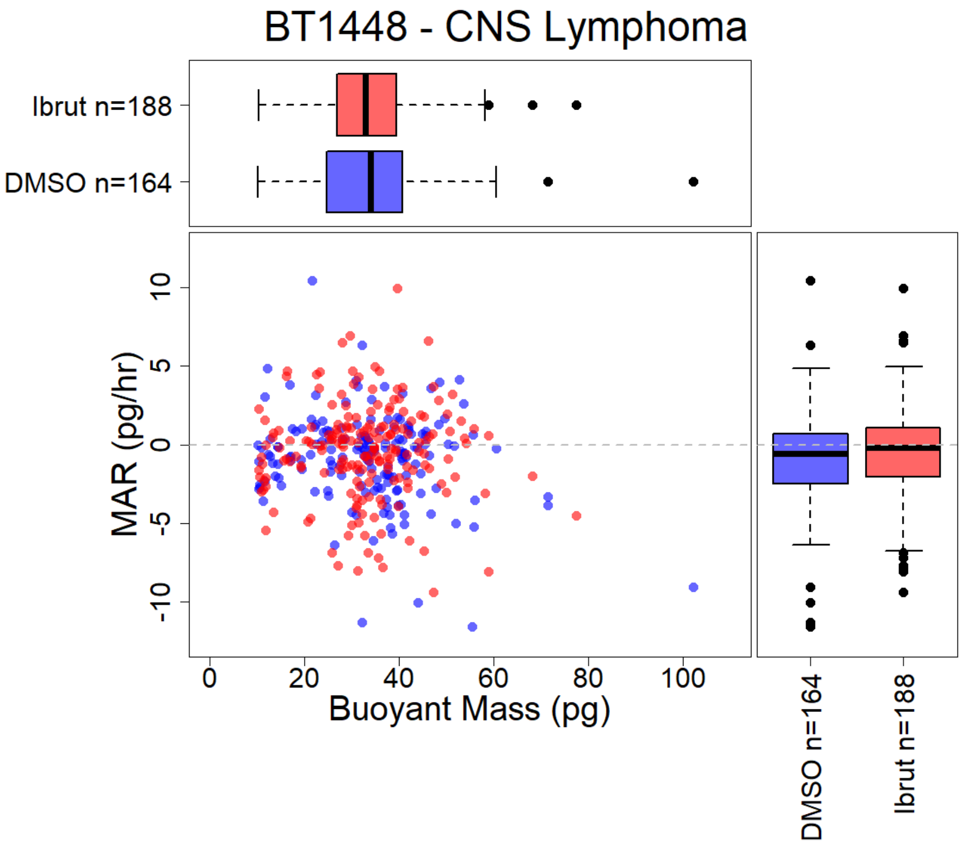

Supplementary Figure 21: Biophysical measurements of BT1448

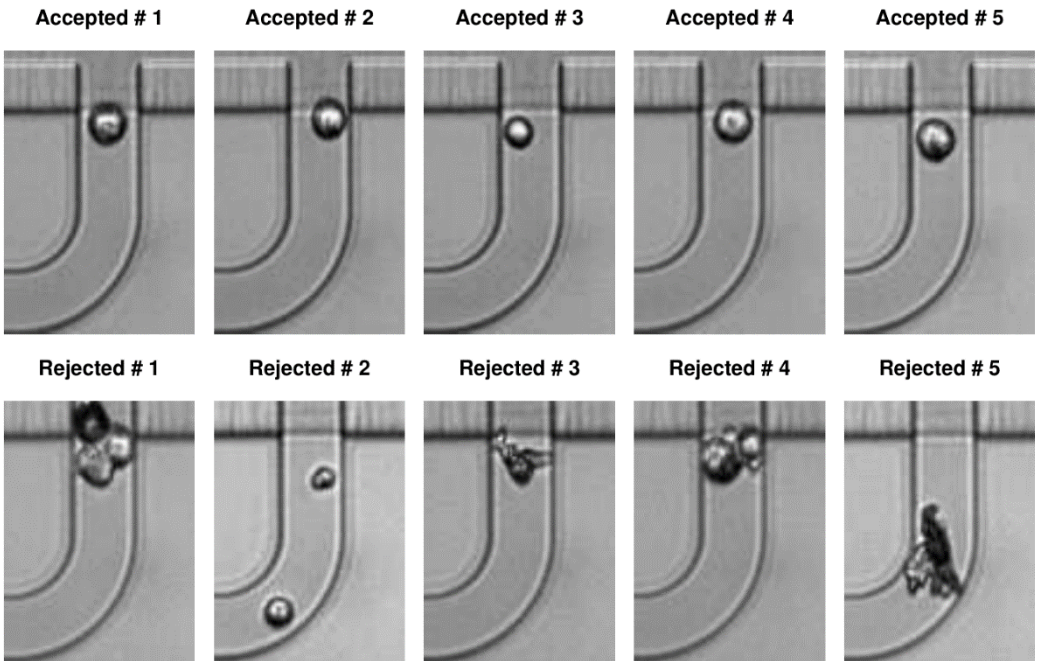

Supplementary Figure 22: Representative images of accepted/rejected cells

SUPLLEMENTARY REFERENCES

1. Cheung, K., Gawad, S. & Renaud, P. Impedance spectroscopy flow cytometry: on-chip label-free cell differentiation. *Cytometry. Part A : the journal of the International Society for Analytical Cytology* **65**, 124-132 (2005).
2. Guck, J. et al. The optical stretcher: a novel laser tool to micromanipulate cells. *Biophysical journal* **81**, 767-784 (2001).
3. Rosenbluth, M.J., Lam, W.A. & Fletcher, D.A. Analyzing cell mechanics in hematologic diseases with microfluidic biophysical flow cytometry. *Lab on a chip* **8**, 1062-1070 (2008).
4. Byun, S. et al. Characterizing deformability and surface friction of cancer cells. *Proceedings of the National Academy of Sciences of the United States of America* **110**, 7580-7585 (2013).
5. Otto, O. et al. Real-time deformability cytometry: on-the-fly cell mechanical phenotyping. *Nature methods* **12**, 199-202, 194 p following 202 (2015).
6. Gossett, D.R. et al. Hydrodynamic stretching of single cells for large population mechanical phenotyping. *Proceedings of the National Academy of Sciences of the United States of America* **109**, 7630-7635 (2012).
7. George, T.C. et al. Distinguishing modes of cell death using the ImageStream multispectral imaging flow cytometer. *Cytometry. Part A : the journal of the International Society for Analytical Cytology* **59**, 237-245 (2004).
8. Wang, X. et al. Enhanced cell sorting and manipulation with combined optical tweezer and microfluidic chip technologies. *Lab on a chip* **11**, 3656-3662 (2011).
9. Dochow, S. et al. Tumour cell identification by means of Raman spectroscopy in combination with optical traps and microfluidic environments. *Lab on a chip* **11**, 1484-1490 (2011).
10. Cermak, N. et al. High-throughput measurement of single-cell growth rates using serial microfluidic mass sensor arrays. *Nat Biotech* **34**, 1052-1059 (2016).
11. Dunn, G.P. et al. Emerging insights into the molecular and cellular basis of glioblastoma. *Genes & development* **26**, 756-784 (2012).
12. Cetin, A.E. et al. Determining therapeutic susceptibility in multiple myeloma by single-cell mass accumulation. *Nature Communications* **8**, 1613 (2017)
13. Filbin, M.G. et al. Developmental and oncogenic programs in H3K27M gliomas dissected by single-cell RNA-seq. *Science* **360**, 331-335 (2018)
14. National Comprehensive Cancer Network. Central Nervous System Cancers Guidelines (Version 1.2018). https://www.nccn.org/profesionals/physician_gls/pdf/cns.pdf. Accessed June 27, 2018
15. Eudocia, Q.L., A Study of Abemaciclib in Recurrent Glioblastoma, *ClinicalTrials.gov*, NCT02981940, Accessed June 27, 2018
16. National Comprehensive Cancer Network. Breast Cancer Guidelines (Version 1.2018). https://www.nccn.org/profesionals/physician_gls/pdf/breast.pdf. Accessed June 27, 2018
17. National Comprehensive Cancer Network. Non-Small Cell Lung Cancer Guidelines (Version 4.2018). https://www.nccn.org/profesionals/physician_gls/pdf/nscl.pdf. Accessed June 27, 2018
18. Grommes, C., Mellinghoff, I., Bruton's Tyrosine Kinase (BTK) Inhibitor, Ibrutinib, in Patients With Refractory/Recurrent Primary Central Nervous System Lymphoma (PCNSL) and Refractory/Recurrent Secondary Central Nervous System Lymphoma (SCNSL), *ClinicalTrials.gov*, NCT02315326, Accessed June 27, 2018
