# Supplementary material for: Microfluidic active loading of single cells enables analysis of complex clinical specimens
Source: Nat Commun. 2018 Nov 14;9:4784. doi: 10.1038/s41467-018-07283-x (PMC6235965; doi:10.1038/s41467-018-07283-x)
Supplement: Supplementary file 2 — Description of Additional Supplementary Files [file 41467_2018_7283_MOESM2_ESM.pdf]

Description of Additional Supplementary Files

File Name: Supplementary Movie 1
Description: Seek to Pinch: the loading channel flow rate switches from high to low when an event is detected in ROI 1.

File Name: Supplementary Movie 2
Description: Queue Cell: secondary particles are delayed by reversing the loading channel flow direction to avoid collision events.

File Name: Supplementary Movie 3
Description: Accept Singlet: morphometric analysis determines the loaded particle is a singlet and allows it to be measured.

File Name: Supplementary Movie 4
Description: Reject Doublet: morphometric analysis determines the particle is a doublet and quickly reverses flow direction of the measurement channel to remove it.

File Name: Supplementary Movie 5
Description: Bead doublet rejection: all active loading components working together to load a bead sample. When a doublet is rejected and removed, the system quickly finds a singlet and loads that in. A second singlet is found, but delayed before loading into the device.
